# Computational Characterization of Unsupported Au(I)···Ir(I) Metallophilic Interactions: Evidence for Strong Dispersive Stabilization

**DOI:** 10.1021/acs.inorgchem.5c03295

**Published:** 2025-08-18

**Authors:** Félix Reboiro, M. Elena Olmos, José M. López-de-Luzuriaga, Miguel Monge

**Affiliations:** Departamento de Química, Instituto de Investigación en Química de la Universidad de La Rioja (IQUR), Madre de Dios 53, 26006 Logroño, La Rioja Spain

## Abstract

Unsupported metallophilic interactions remain experimentally unexplored in gold–iridium heterobimetallic complexes. To address this gap, we conducted a computational study on a series of model systems of the form [Ir­(CO)­X­(PH_3_)_2_]­[AuR_2_], where X = Cl, Br, and I and R = H, CH_3_, NH_3_, and PH_3_, to evaluate their strength and feasibility. Optimizations were performed at the MP2/def2-TZVPP level of theory, and interaction energies at equilibrium distances were assessed through potential energy curves (PECs). To account for relativistic effects, calculations were complemented by spin-component-scaled (SCS)-MP2 within the zero-order regular approximation (ZORA). Further insight into the nature of these interactions was gained through analyses of NBO effective charges, bond orders, natural energy decomposition analysis (NEDA), and topological examination of electron density using QTAIM and IGMH frameworks. Our results revealed intermetallic distances consistent with significant noncovalent attraction, exhibiting unexpectedly high interaction energy values (20–60 kJ mol^–1^). The presence of a bond critical point (BCP) along the Au­(I)···Ir­(I) bond path across all model systems confirms the attractive character of these interactions, which are characterized as regular closed-shell with a partial degree of electron sharing. Overall, this study underscores the potential importance of such noncovalent interactions in the rational design of unsupported gold–iridium heterobimetallic complexes with promising properties.

## Graphical abstract

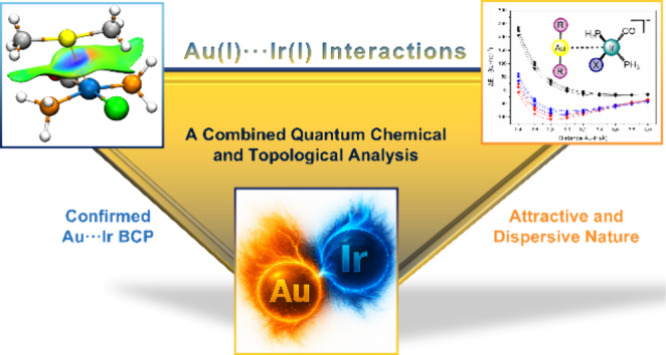

## 1 Introduction

Over the past three decades, metallophilic interactions (i.e., attractive forces between closed-shell metal cations such as d^10^, d^8^, and d^10^s^2^ electron systems)^1^ have garnered considerable attention from both experimental and theoretical perspectives to better understand their fundamental characteristics.^2^ These weak noncovalent interactions, particularly involving Au­(I) cation, play a key role in stabilizing reactive catalytic intermediates,^3^ and contribute to phenomena such as supramolecular assembly,^4^ mechanochromism,^5^ vapochromism,^6^ and luminescence.^7^

Metallophilicity encompasses both homometallic^8^ and heterometallic^9^ interactions, typically characterized by van der Waals-type forces in the range of 15 to 50 kJ mol^–1^.^10^ These interactions are primarily driven by dispersion forces and are significantly enhanced by relativistic effects in heavy metal cations.^11^ Although intermetallic distances shorter than the sum of van der Waals radii are commonly used as indicators of metallophilicity,^12^ variability in reported radii limits this as a definitive criterion.^13^ Numerous computational studies employing density functional theory (DFT) and post-Hartree–Fock (HF) methods, such as MP2, spin-component-scaled (SCS)-MP2, or CCSD­(T), have been performed, yet the precise nature of metallophilic interactions remains an open question.^14^

Gold-containing heterobimetallic compounds have attracted considerable attention due to the tunable nature of their metallophilic interactions,^15^ and their potential to enhance the efficiency of monometallic catalysts by enabling alternative pathways for bond activation.^16^ While homometallic systems involving Au­(I)···Au­(I) contacts are well established and frequently observed as recurring structural motifs,^5a,8a^ reports of heterobimetallic systems displaying Au­(I)···Ir­(I) interactions remain comparatively scarce. The structural design of dinuclear gold–iridium heterobimetallic complexes has commonly involved two bridging ligands that position the metal centers in proximity (Figure 1A). This structural framework is inherently unsymmetrical; thus, it is expected to exhibit different properties from those of its homometallic counterparts.

**Figure 1:**
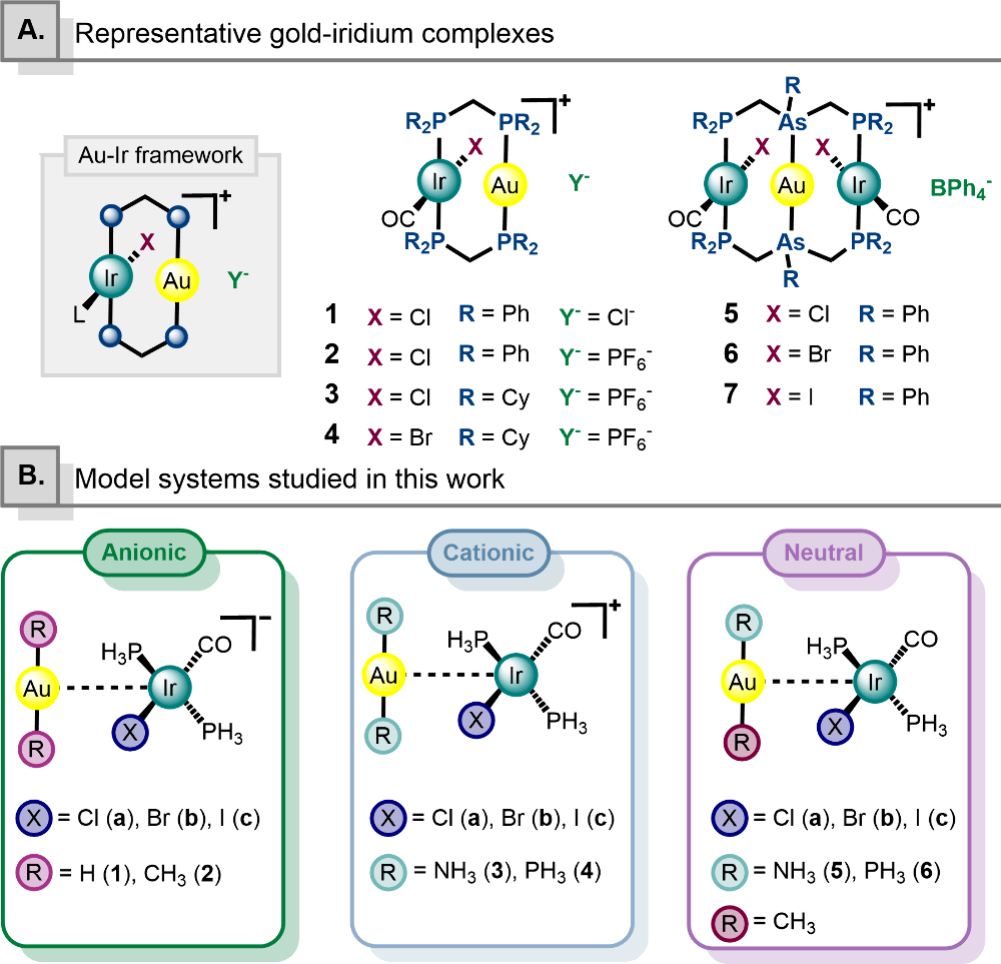
(A) Representative supported gold–iridium complexes in the literature. (B) Computational model systems employed in this work.

The first complex of this type was reported by Shaw and co-workers: [Ir­(CO)­Cl­(μ-dppm)_2_Au]Cl (dppm = bis­(diphenylphosphino)­methane), which features a bridging dppm ligand connecting gold and iridium centers (see Figure 1, complex **A1**).^17^ Subsequently, Balch and co-workers reported the structure and photoluminescence properties of the related complex [Ir­(CO)­Cl­(μ-dppm)_2_Au]­PF_6_ (see Figure 1, complex **A2**).^18^ The Au–Ir distance in their X-ray structure is 2.986(1) Å, which exceeds the typical range observed for metal–metal bonding. The emission spectrum displayed two distinct bands: one attributed to fluorescence, and the other, at lower energy, to phosphorescence. Additionally, an in-depth investigation of these luminescent properties was later carried out through quantum chemical calculations.^19^ Building on this framework, Nocera and co-workers synthesized related complexes by modifying the bridging ligand, yielding compounds of the form [Ir­(CO)­X­(μ-dcpm)_2_Au]­PF_6_ (dcpm = bis­(dicyclohexylphosphino)­methane; X = Cl, Br), shown in Figure 1 as complexes **A3** and **A4**, respectively.^20^ Notably, these compounds can be oxidized with halogen to afford bimetallic Au­(II)–Ir­(II) species.

Other representative examples of Au–Ir frameworks are the metallamacrocyclic complexes [Ir_2_(CO)_2_X_2_(μ-dpma)_2_Au]­BPh_4_ (dpma = bis­(diphenylphosphino)­methylphenylarsine; X = Cl, Br, I), reported by Balch and co-workers, and depicted in Figure 1 as complexes **A5–A7**, respectively.^21^ The X-ray structure of complex **A5** revealed a nearly linear trinuclear Ir–Au–Ir chain with Au–Ir distances of 3.012(1) and 3.059(1) Å, which are relatively long for bridged Au–Ir bonds. All complexes exhibited emissive behavior, tentatively assigned to fluorescence, and displayed similar spectral profiles. The substitution of the halide resulted in small red shifts in the emission, following the trend Cl < Br < I. Despite the numerous well-characterized examples of supported gold–iridium heterobimetallic complexes and their potential applications, gold–iridium systems featuring unsupported metallophilic interactions have, to the best of our knowledge, not yet been reported.

Our research group has extensive experience studying complexes bearing metallophilic interactions through both experimental and computational approaches.^22,23^ Accordingly, we carried out a comprehensive computational and topological investigation at the correlated MP2 level of theory on model systems exhibiting unsupported Au­(I)···Ir­(I) interactions (Figure 1B). The goal of this study is to assess the intrinsic strength and feasibility of these interactions in different electronic systems, thereby offering valuable insights to guide the design of future heterobimetallic complexes for experimental work.

## 2 Computational Details

Full optimizations were performed at the MP2 ^24^ and RHF^25^ levels of theory using the Gaussian 16 software package.^26^ The Karlsruhe def2-TZVPP basis sets^27^ were employed for all atoms in combination with 60-electron effective core potentials (def2-ECPs)^28^ for gold and iridium atoms. Frequency analyses at the same level of theory confirmed that the optimized structures correspond to a true minimum (no imaginary frequencies were detected). Molecular structures were visualized and rendered using GaussView^29^ and UCSF ChimeraX.^30^

Relativistic effects in model systems **a** were investigated using the zero-order regular approximation (ZORA)^31^ as implemented in the ORCA 5 program.^32^ The model systems were fully optimized at the SCS-MP2-ZORA level of theory,^33^ employing SARC-ZORA-TZVPP basis sets for the metal atoms and ZORA-SVP basis sets for all other atoms.^34^ Counterpoise-corrected interaction energies were calculated using SARC-ZORA-TZVPP basis sets for the metal atoms and ZORA-TZVPP basis sets for the remaining atoms. Nonrelativistic ZORA calculations were carried out by setting the inverse fine-structure constant to 100*c*, where *c* is the speed of light in vacuum. For standard ZORA calculations, the RIJCOSX approximation was employed.^35^ These two approaches are denoted as SCS-MP2-ZORA (standard) and SCS-MP2-ZORA^NR^ (nonrelativistic). The superposition of structures was performed using the VMD software package.^36^

Interaction energies (Δ*E*_int_) were obtained at the MP2, RHF, and PBE0-D3BJ^37^ levels of theory with def2-TZVPP basis sets employing the counterpoise correction (cp) for the basis set superposition error (BSSE),^38^ see eq S1. Potential energy curves (PECs) were fitted using the four-parameter Herschbach–Laurie function, see eq S2.^39^ Counterpoise-corrected interaction energies were further refined at the SCS-MP2 and DLPNO–CCSD­(T)^40^ levels of theory employing def2-TZVPP basis sets, def2-ECPs, and the RIJCOSX approximation using ORCA 5.

Natural bonding orbital (NBO)^41^ and Wiberg bond order (WBO)^42^ calculations were performed using the Gaussian 16 package. The intrinsic bond strength Index (IBSI)^43^ and fuzzy bond order (FBO)^44^ were computed with Multiwfn software,^45^ at the MP2/def2-TZVPP level of theory. Natural energy decomposition analysis (NEDA)^46^ was conducted using Gaussian 16 and NBO 7.0 ^47^ at the DFT hybrid functional PBE0 with D3­(BJ) empirical dispersion correction,^37^ employing def2-TZVPP basis sets.

Topological analyses of the MP2/def2-TZVPP electron density were carried out using the quantum theory of atoms in molecules (QTAIM)^48^ and the independent gradient model based on Hirshfeld partitioning (IGMH),^49^ as implemented in Multiwfn software. Electron density visualizations were rendered with the VMD software package. Further computational details and equations are available in the Supporting Information.

## 3 Results and Discussion

### 3.1 Model Systems and Structure Optimization

Based on experimental complexes that employ bridging ligands to position gold and iridium centers in proximity (see Figure 1A), we designed simplified model systems inspired by these complexes, featuring geometries suitable for the potential establishment of metallophilic interactions. The resulting models consist of a square-planar Ir­(I) fragment of formula [Ir­(CO)­X­(PH_3_)_2_], where X = Cl (**a**), Br (**b**) or I (**c**), analogous to the classic Vaska complex,^50^ and a linear Au­(I) fragment of the type [AuR_2_]. These geometries are, in principle, well-suited to promote the formation of targeted metallophilic interactions.

Therefore, we investigated a series of model systems to explore how Au­(I)···Ir­(I) interactions are affected by the overall charge of the complex, the nature of the ligands coordinated to the gold center, and the variation of the halide in the iridium fragment. The studied model systems include anionic species of the type {[AuR_2_]­[Ir­(CO)­X­(PH_3_)_2_]}^−^ where R = H (**1**) or CH_3_ (**2**), cationic analogues {[AuR_2_]­[Ir­(CO)­X­(PH_3_)_2_]}^+^ where R = NH_3_ (**3**) or PH_3_ (**4**), and neutral systems of the form [Au­(CH_3_) R]­[Ir­(CO)­X­(PH_3_)_2_] where R = NH_3_ (**5**) or PH_3_ (**6**).

First, all model systems were fully optimized at the MP2/def2-TZVPP level of theory without geometry constraints. The optimized structures are shown in Figure 2, and a selection of bond lengths and angles is listed in Table S1 (see the Supporting Information).

**Figure 2:**
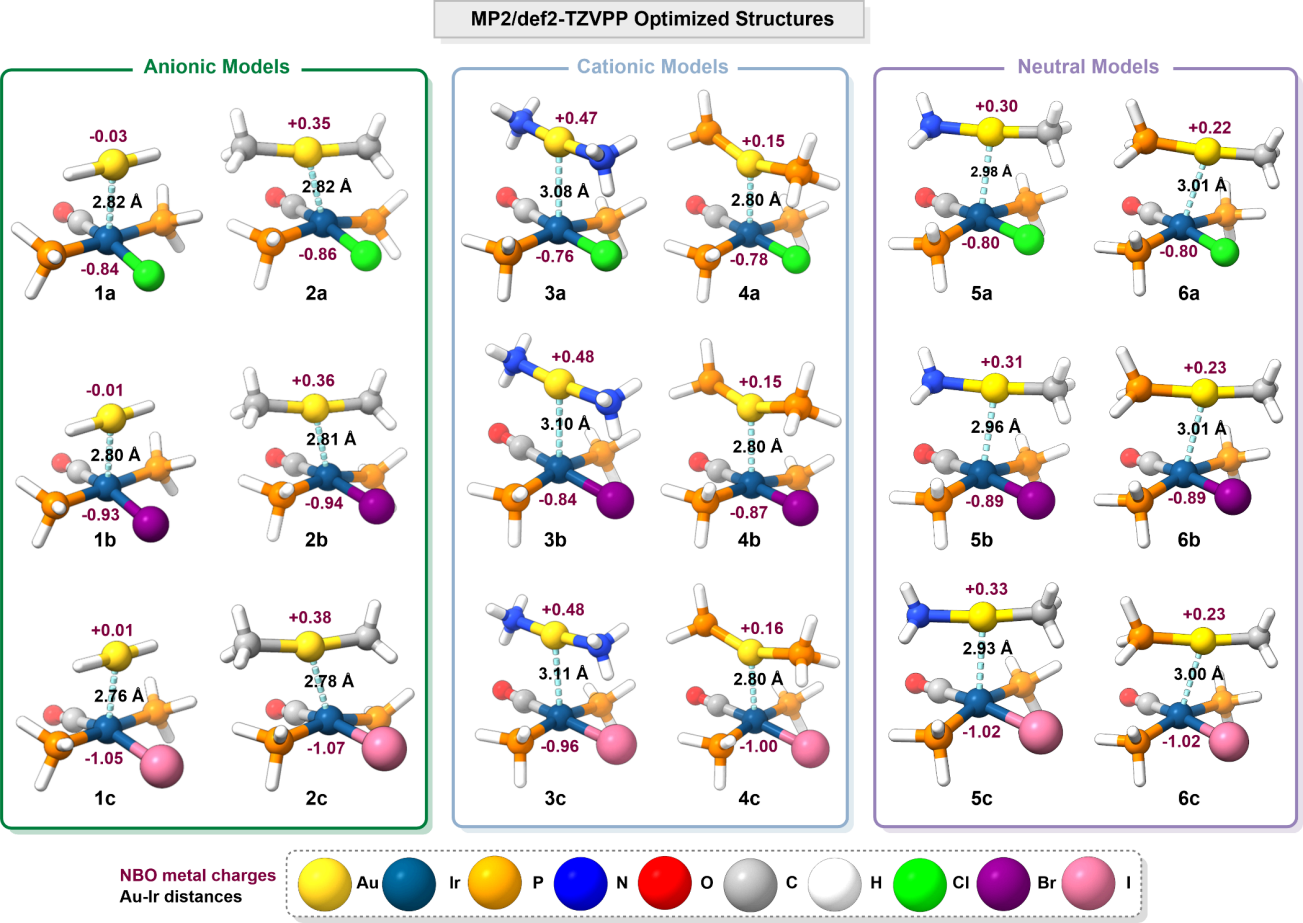
Representations of the MP2/def2-TZVPP optimized structures of model systems **1**–**6**. The most relevant distances and the NBO metal charges, calculated at the same level of theory as the optimizations, are included.

All optimized structures reached a minimum, with both fragments positioned in nearly parallel planes that enabled interaction between the metal centers, adopting an almost eclipsed arrangement. The Au–Ir distances are surprisingly short for unsupported interactions, ranging from 2.76 to 3.11 Å, all below the sums of their respective van der Waals radii (i.e., 3.66 Å,^13a^ 4.10 Å,^13b^ and 4.73 Å,^13c^ depending on the chosen reference), yet remain too long to be considered indicative of a metallic bond, given that the sum of their corresponding metallic radii is 2.60 Å.^51^ These values suggest a potentially attractive interaction between the metal centers; however, intermetallic distance alone is not sufficient to definitively confirm the presence of a metallophilic interaction.^12^ Moreover, the NBO charges on the metal centers indicate that the iridium atom bears a higher electron density than the gold center.

In addition, the same set of structures was fully optimized at the RHF/def2-TZVPP level of theory without any geometrical constraints. The resulting geometries are presented in Figure 3 and summarized in Table S1.

**Figure 3:**
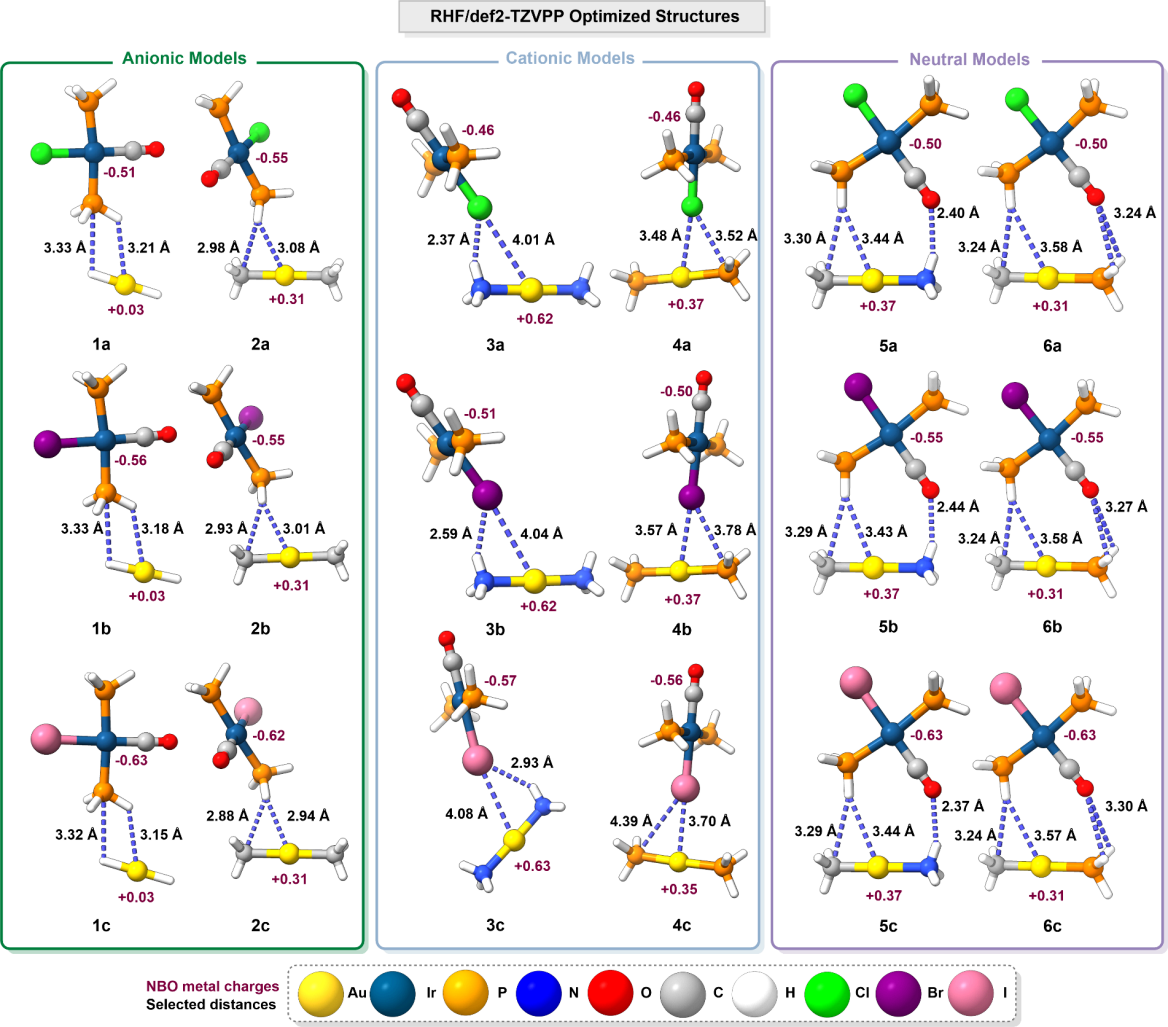
Representations of the RHF/def2-TZVPP optimized structures of model systems **1**–**6**. The most relevant distances and the NBO metal charges, calculated at the same level of theory as the optimizations, are included.

When these structures are compared to those optimized at the MP2 level of theory, the metallophilic interactions are no longer present and alternative noncovalent contacts emerge, primarily involving hydrogen or halogen bonding with the gold fragment. Anionic model systems **1** form P···H–Au and Au···H–P contacts, aligning the fragments in a coplanar arrangement. In contrast, model systems **2** exhibit two hydrogen bonds involving P–H···Au and C–H···P interactions. In the cationic model systems **3** and **4**, two halogen bonds are present: N–H···X, P–H···X, and X···Au. In model systems **3**, the former interaction is predominant, whereas in model systems **4**, the latter dominates. In neutral model systems **5** and **6**, both P–H···C and P–H···Au interactions are identified, contributing to a coplanar arrangement of the fragments. Additionally, model system **5** exhibits a single R–H···O=C interaction, whereas two such interactions are observed in model system **6**. Furthermore, NBO analysis reveals a depletion of electron density at both metal centers under this level of theory compared to the MP2 one.

Upon reoptimization of the RHF-optimized structures at the MP2 level of theory, they converged to the same minima as those obtained from the initial MP2 optimizations. This confirms that, at the MP2 level of theory, the true minima correspond to the structures shown in Figure 2. These results highlight the importance of incorporating electron correlation for an accurate description of metallophilic interactions that are predominantly dispersive in nature.

However, it is important to emphasize that while all dispersion interactions arise from electron correlation, not all correlation effects correspond to dispersion.^2^ This distinction is particularly relevant when interpreting interaction energies computed using correlated methods, such as MP2. In contrast, methods that neglect electron correlation, such as the Hartree–Fock (HF) method, typically fail to capture dispersive interactions and may instead favor alternative interaction motifs such as hydrogen or halogen bonding. These types of interactions are favored due to their stronger electrostatic component compared to metallophilic ones.^23a^ Considering this, a detailed computational investigation of the Au­(I)···Ir­(I) interactions was carried out using the MP2-optimized structures.

### 3.2 Role of Relativistic Effects

Full optimizations of model systems **a** were carried out at both the SCS-MP2-ZORA and SCS-MP2-ZORA^NR^ levels of theory (see the Section 2). The zero-order regular approximation (ZORA) was employed and compared to its nonrelativistic counterpart (ZORA^NR^) to assess the role of relativistic effects in the formation of metallophilic interactions. Given that the MP2 method is known to overestimate metallophilic interactions,^11^ we employed the SCS-MP2 level of theory, which provides a reliable and balanced efficient description of electron correlation, delivering results in good agreement with those obtained from the more demanding CCSD­(T) method.^52^ Additionally, the ZORA formalism was used to incorporate relativistic effects at the all-electron level, in contrast to ECPs, which approximate these effects only for the valence electrons. Thus, the superimposed optimized structures are shown in Figure 4, and a selection of bond lengths and angles is provided in Table S2.

**Figure 4:**
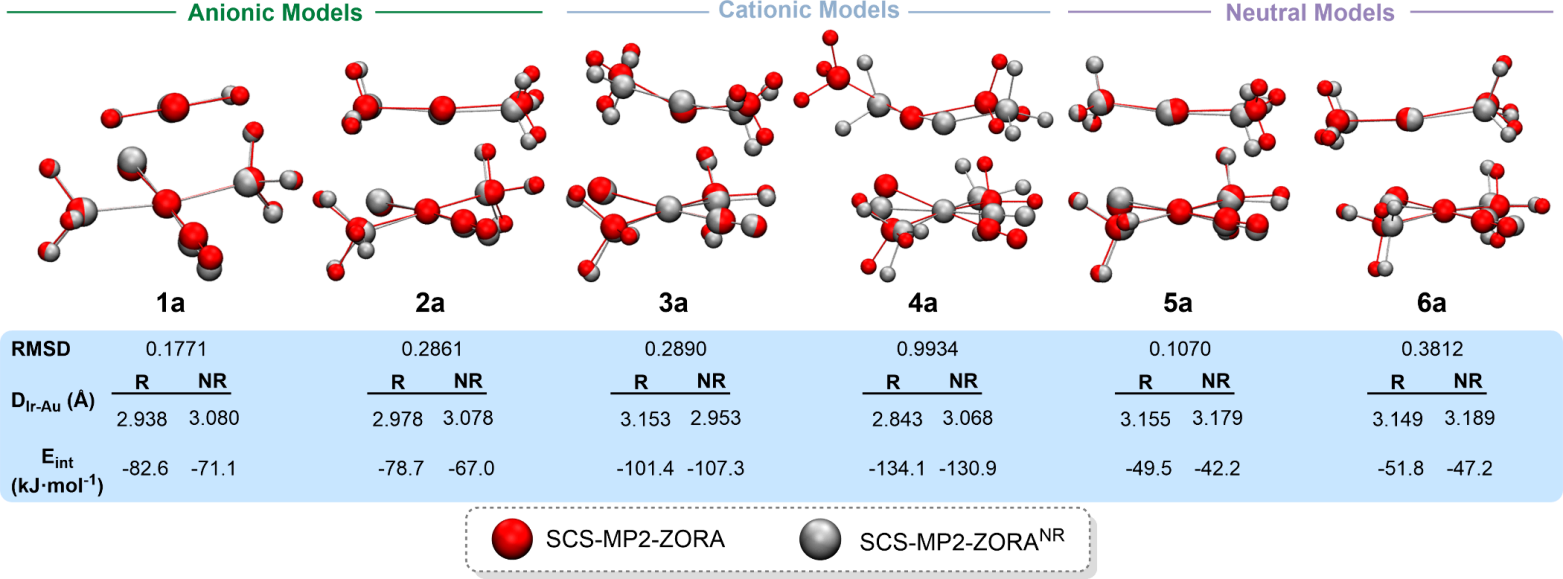
Representations of the superimposed optimized structures at the SCS-MP2-ZORA and SCS-MP2-ZORA^NR^ level of theory for models **a**.

The SCS-MP2 optimized structures exhibit good agreement with those obtained at the MP2 level. The Au–Ir distances are slightly longer in the SCS-MP2 results, which is consistent with the known tendency of MP2 to overestimate the strength of such interactions. Therefore, the model systems are well described by this more accurate and efficient electron correlation method, validating the reliability of the MP2 optimized structures.

Upon comparison of the superimposed SCS-MP2-ZORA and SCS-MP2-ZORA^NR^ structures, it is observed that, as expected, the metal–metal distances generally increase slightly in the absence of relativistic effects, apart from model system **3a**. This deviation in model system **3a** may be attributed to N–H···X or N–H···O=C interactions, which could be more favored under the nonrelativistic approach and contribute to shortening the Au–Ir distance. The root-mean-square deviation (RMSD), which quantifies the average positional difference between the corresponding atoms in two superimposed molecular structures, shows low values ranging from approximately 0.1 to 0.38 Å for most model systems, indicating structural similarity. However, model system **4a** exhibits a higher RMSD value of 0.99 Å, suggesting greater structural deviation in the [Au­(PH_3_)_2_]^+^ fragment. As expected, the inclusion of relativistic effects at the SCS-MP2-ZORA level of theory results in cp interaction energies slightly higher than those calculated at the SCS-MP2-ZORA^NR^ level of theory. The only exceptions are model systems **3**, where the metal centers are closer in the ZORA^NR^ structure than in the relativistic ZORA one. This deviation is likely due to enhanced stabilization through hydrogen bonding in the nonrelativistic approach, as previously discussed.

Hence, the results indicate that relativistic effects tend to favor the formation of metallophilic interactions. However, as suggested by the low RMSD and interaction energy values, their contribution represents only about 2 to 15% of the total interaction energy, significantly lower than in systems involving Au­(I)···Au­(I) or Au­(I)···Hg­(II) interactions, where relativistic effects account for approximately 22–27% of the total interaction energy.^1a,23b^ This observation is consistent with the weaker relativistic character of the Ir­(I) cation compared to Au­(I) or Hg­(II).^53^ Consequently, these effects are also expected to be comparable to those observed in systems involving Au­(I)···Pt­(II) or Au­(III)···Au­(III) interactions.^23e,23f^

### 3.3 Potential Energy Curves

Potential energy curves (PECs) were computed by varying the Au–Ir distance in the MP2-optimized structures while keeping the remaining geometry fixed. Interaction energies (Δ*E*_int_) were calculated at MP2, RHF, and PBE0-D3­(BJ) levels of theory employing the def2-TZVPP basis sets. To correct the BSSE, a counterpoise correction was applied at each point, and the resulting data were fitted using the Herschbach-Laurie four-parameter function. Thus, Figure 5 displays the resulting PECs, with the raw data provided in Tables S3–S8. Additional computational details and equations are provided in the Supporting Information.

**Figure 5:**
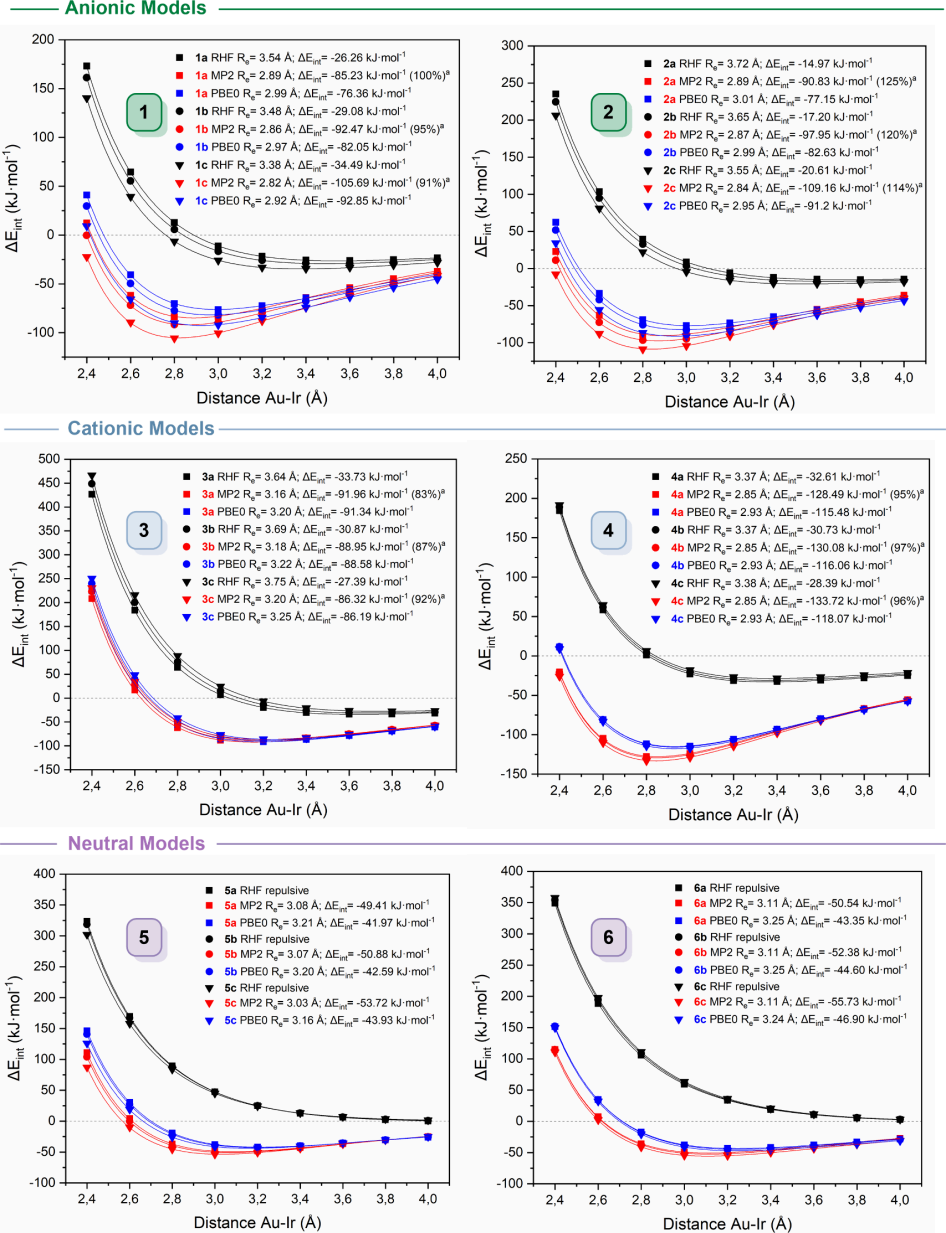
Total interaction energy as a function of the Au–Ir distance calculated at the RHF, MP2, and PBE0-D3­(BJ) levels of theory. Raw data are provided in the Supporting Information, Tables S3–S8. ^a^ Electron correlation contribution was calculated employing eq S3.

Analysis of the shapes of the PECs provides valuable insights into the characteristics of the interactions within the model systems. When two metal centers come closer, the interaction energy becomes more negative, reaching a minimum at an optimal distance, balancing attractive and repulsive forces. Beyond this distance, the interaction weakens and approaches zero as the metals separate further. Thus, their attractive or repulsive nature can be assessed by analyzing the presence or absence of a minimum in the PECs. Thus, a minimum indicates attraction, while its absence suggests repulsion. Furthermore, dispersion forces can be estimated by evaluating how the electron correlation is incorporated within the computational framework. Since electron correlation is neglected at the RHF level of theory, it fails to adequately describe dispersion-driven interactions, including metallophilic contacts and hydrogen and halogen bonding. Conversely, correlated methods like MP2 and dispersion-corrected DFT functionals such as PBE0-D3­(BJ) explicitly account for these phenomena, providing a more accurate treatment of such interactions.

The MP2 level of theory captures dispersion interactions inherently by including electron correlation through a second-order perturbative treatment. However, it is well-known that MP2 tends to overestimate the strength of dispersion interactions.^1a^ Despite this tendency, MP2 is still considered a reliable tool for identifying the presence of dispersion forces.^11b^ On the other hand, the PBE0 functional does not inherently describe long-range dispersion and therefore requires empirical corrections. Grimme’s D3­(BJ) correction addresses this limitation by introducing atom-pairwise dispersion terms modulated by damping functions and fitted parameters to approximate long-range interactions more accurately. Accordingly, the contribution of electron correlation was quantified as the difference in interaction energies computed at the MP2 and RHF levels of theory (eq S3).

The calculated PECs for all model systems confirm the attractive nature of the Au­(I)···Ir­(I) interactions with well-defined minima observed at all levels of theory for both anionic and cationic model systems. For anionic model systems **1** and **2**, equilibrium distances at the MP2 level of theory range from 2.82 to 2.89 Å, with associated interaction energies between −85 and −106 kJ mol^–1^ for model systems **1**, and −91 to −109 kJ mol^–1^ for model systems **2**. Similarly, cationic model systems **3** and **4** exhibit minima across all levels of theory, too. At the MP2 level of theory, model systems **3** shows the largest equilibrium distances (3.10 to 3.20 Å) across all model systems, and interaction energies ranging from −86 to −92 kJ mol^–1^, consistent with a weaker Au­(I)···Ir­(I) interaction. In contrast, model systems **4** presents the shortest distance (2.85 Å) among the cationic systems and the highest interaction energies (−128 to −134 kJ mol^–1^) across all of the studied models. This suggests that PH_3_ ligands provide the most effective stabilization of the metallophilic interaction, likely due to their superior electron-donating ability toward the gold center. For neutral model systems **5** and **6**, PECs computed at the RHF level exhibit no minima, indicating pure repulsion in the absence of electron correlation effects. However, at the MP2 level of theory, both model systems show equilibrium distances within 3.03–3.11 Å, with interaction energies of −49 to −54 kJ mol^–1^ for model systems **5** and −51 to −56 kJ mol^–1^ for model systems **6**. Although less stabilized than their charged counterparts, these values still reflect significant noncovalent attraction dominated by dispersion forces.

Therefore, the MP2 equilibrium distances are significantly shorter than the sum of the van der Waals radii of the metal atoms, and the corresponding interaction energies, approximately 50 kJ mol^–1^ for neutral model systems and ranging from 90 to 125 kJ mol^–1^ for the ionic ones, fall within the typical range for noncovalent interactions. A clear correlation is observed between the Au–Ir distance and the interaction energy: the shorter the distance at the minimum, the more stabilizing the interaction becomes. Substitution across the halogen series results in an increase in interaction energy following the trend Cl (**a**) < Br (**b**) < I (**c**) for all model systems. However, this increase is modest, ranging from 1 to 10 kJ mol^–1^.

The significance of electronic correlation is demonstrated by the shorter intermetallic distances and enhanced stabilization observed at the MP2 level of theory compared to the RHF level of theory. For all PECs, electron correlation effects are essential for stabilization (83 to 125%), highlighting the dispersive nature of the interactions. In neutral model systems, neglecting the correlation leads to purely repulsive behavior, consistent with the absence of electrostatic contributions. In contrast, ionic model systems benefit not only from dispersion but also from significant Coulombic forces, further enhancing their stability. However, it is important to note that these energies are not solely due to metallophilic interactions; secondary contacts, such as hydrogen bonding with the NH_3_ ligand in model systems **3**, also contribute significantly to the total stabilization.

Furthermore, the PECs calculated at the PBE0-D3­(BJ) level of theory are in close agreement with those from MP2 calculations, although the interaction energies are systematically lower by about 10 kJ mol^–1^. All equilibrium distances predicted by the PBE0-D3­(BJ) level of theory are longer than those at the MP2 level of theory, indicating the expected weaker attraction in the former. An important consideration is that DFT functionals can suffer from delocalization error (DE),^54^ which significantly impacts physical and chemical properties. However, hybrid functionals such as PBE0, which include a fraction of the exact exchange, effectively reduce this error. Consequently, the similarity to the typically overestimated MP2 results can be attributed to this mitigation of delocalization error, offering reliable results at a substantially lower computational cost.

To improve the accuracy of the computed interaction energies, single-point cp-energy calculations were performed at the MP2-PECs minima for each model system using more sophisticated and demanding methods. This approach enables a comparative assessment of the MP2 level of theory against more advanced approaches such as SCS-MP2 and DLPNO–CCSD­(T), which offer a more accurate treatment of electron correlation. The results, summarized in Table 1, illustrate the deviations from the MP2 level of theory and provide insight into the reliability of each method in describing the dispersive nature of Au­(I)···Ir­(I) interactions.

**Table 1:** Counterpoise Corrected Interaction Energies (kJ mol^–1^) Computed at SCS-MP2 and DLPNO–CCSD­(T) Levels of Theory Employing def2-TZVPP Basis Sets and def2-ECPs at the MP2 Minima of the Potential Energy Curves (Å)

model	*R*_Au–Ir_	Δ*E*_int_^SCS‑MP2^	Δ*E*_1_ ^a^	Δ*E*_int_^CCSD(T)^	Δ*E*_2_ ^b^
**1a**	2.89	–76.60	+8.63	–57.17	+28.06
**1b**	2.86	–84.20	+8.27	–65.73	+26.74
**1c**	2.82	–96.40	+9.29	–75.74	+29.95
**2a**	2.89	–82.47	+8.36	–63.12	+27.71
**2b**	2.87	–89.29	+8.66	–69.40	+28.55
**2c**	2.84	–100.49	+8.67	–79.15	+30.01
**3a**	3.16	–91.63	+0.33	–79.27	+12.69
**3b**	3.18	–89.57	–0.62	–88.95	+12.00
**3c**	3.20	–86.97	–0.65	–72.94	+13.38
**4a**	2.85	–119.50	+8.99	–95.84	+32.65
**4b**	2.85	–120.60	+9.48	–95.54	+34.54
**4c**	2.85	–123.67	+10.05	–95.83	+37.89
**5a**	3.08	–45.20	+4.21	–29.56	+19.85
**5b**	3.07	–46.29	+4.59	–30.36	+20.52
**5c**	3.03	–48.95	+4.77	–31.71	+22.01
**6a**	3.11	–45.72	+4.82	–30.57	+19.97
**6b**	3.11	–47.37	+5.01	–31.28	+21.10
**6c**	3.11	–50.54	+5.19	–33.23	+22.50

a Δ*E*_1_ represents the energy difference between SCS-MP2 and MP2/def2-TZVPP calculations (kJ mol^–1^).

b Δ*E*_2_ represents the energy difference between DLPNO–CCSD­(T) and MP2/def2-TZVPP calculations (kJ mol^–1^).

As expected, the computed interaction energies are lower (Δ*E* > 0) than those computed at the MP2 level of theory, reflecting the improved accuracy and efficiency in the treatment of electron correlation compared to the MP2 level of theory, which tends to overestimate this type of interaction. The gold-standard CCSD­(T) method yields substantially lower interaction energies (Δ*E* = 20–30 kJ mol^–1^) than those obtained with the SCS-MP2 level of theory (Δ*E* = 5–10 kJ mol^–1^). Nevertheless, all interactions remain attractive and fall within the energy range characteristic of noncovalent interactions. These findings are consistent with the trends observed in the MP2 potential energy curves analysis.

Consequently, we consider the MP2 level of theory appropriate for studying metallophilic interactions in our computational model systems, as it produces results consistent with those from higher-level methods. Therefore, all subsequent calculations were performed by using the geometries derived from the MP2 minima at the PECs.

### 3.4 Bond Parameters and Energy Decomposition Analysis

To gain further insight into the nature of the Au­(I)···Ir­(I) interactions, several analyses of different parameters were performed. Effective natural bond orbital (NBO) charges were calculated at the minimum MP2-PECs for all model systems. The NBO metal charges are summarized in Table 2, while additional relevant values are listed in Table S9.

**Table 2:** Effective NBO Metal Charges and Bond and Penetration Indices for the Metallophilic Interactions Calculated at the MP2/def2-TZVPP Level of Theory

model	Au *q* ^NBO^ ^a^	Ir *q* ^NBO^ ^a^	WBO	IBSI	FBO	*p*_Au–Ir_
**1a**	–0.03 (−0.15)	–0.82(−0.65)	0.32	0.069	0.74	93.7
**1b**	–0.02 (−0.15)	–0.91 (−0.72)	0.34	0.073	0.77	95.0
**1c**	–0.01 (−0.15)	–1.03 (−0.82)	0.36	0.080	0.82	97.2
**2a**	+0.34 (+0.20)	–0.84(−0.65)	0.30	0.071	0.64	93.5
**2b**	+0.35 (+0.20)	–0.92 (−0.72)	0.31	0.074	0.66	94.5
**2c**	+0.37 (+0.20)	–1.05 (−0.82)	0.33	0.080	0.71	96.3
**3a**	+0.47 (+0.47)	–0.75 (−0.65)	0.17	0.034	0.45	79.4
**3b**	+0.48 (+0.47)	–0.83 (−0.72)	0.17	0.033	0.44	78.0
**3c**	+0.48 (+0.47)	–0.95 (−0.82)	0.16	0.032	0.43	76.9
**4a**	+0.15 (+0.23)	–0.78 (−0.65)	0.27	0.075	0.75	95.7
**4b**	+0.16 (+0.23)	–0.86 (−0.72)	0.27	0.076	0.76	95.7
**4c**	+0.16 (+0.23)	–0.99 (−0.82)	0.28	0.077	0.76	96.0
**5a**	+0.29 (+0.23)	–0.77 (−0.65)	0.22	0.043	0.50	88.8
**5b**	+0.30 (+0.23)	–0.86 (−0.72)	0.23	0.045	0.51	89.7
**5c**	+0.32 (+0.23)	–1.00 (−0.82)	0.25	0.051	0.55	91.6
**6a**	+0.22 (+0.22)	–0.78(−0.65)	0.19	0.041	0.43	86.4
**6b**	+0.22 (+0.22)	–0.86 (−0.72)	0.19	0.042	0.43	87.9
**6c**	+0.22 (+0.22)	–0.99 (−0.82)	0.19	0.044	0.43	87.9

a The charges of the metal atoms in their respective optimized monomers are shown in parentheses.

The effective NBO charge distribution across model systems indicates that gold atoms carry significantly lower charge (−0.01 to +0.48) than their formal +1 oxidation state. In contrast, the iridium atom exhibits a notable accumulation of electron density, with effective charges ranging from approximately −0.75 to −1.05, indicating substantial electron donation from the surrounding ligands that effectively lowers its formal +1 oxidation state. When compared to their respective monomeric fragments, the gold atom exhibits an increase in positive charge (apart from model systems **4**), while the iridium atom shows a corresponding decrease in negative charge. This shift indicates electron donation from the gold fragment to the iridium fragment, consistent with a donor–acceptor character. As expected, the effective charge on iridium atoms decreases from chlorine to iodine model systems (**a** to **c**), reflecting the increasing polarization of the halide ligand. As a result, the electron density is progressively transferred from the Au­(I) fragment to the Ir­(I) fragment, and the enhanced charge separation strengthens the Coulombic attraction, contributing to the stabilization of the metallophilic interaction. However, in model systems **4**, an increase in electron density is observed on both metal centers, due to the phosphine ligands donating electron density more effectively to the gold atom (the only positively charged ligand, see Table S9). This facilitates a bidirectional charge transfer mechanism, analogous to that previously reported for Au­(I)···Hg­(II) interactions.^22a^ As a result, these model systems exhibit enhanced stabilization, yielding the highest interaction energy among all of the systems analyzed.

To further assess the strength of the metallophilic interactions, we computed the Wiberg bond order (WBO), intrinsic bond strength index (IBSI), and Fuzzy bond order (FBO), each based on a distinct portioning of the molecular space to quantify electron sharing and delocalization between the metal centers (Table 2). These dimensionless indices measure the extent of electron pairs shared between atomic basins and serve as reliable indicators of electron delocalization in closed-shell systems like those studied here. The consistently low values across all indices confirm that the Au­(I)···Ir­(I) interactions are noncovalent, supporting their predominantly dispersive nature. However, FBO values are comparatively higher, suggesting some degree of electron sharing between the metal centers due to the short Au–Ir distances, especially in model systems **1**, **2**, and **4**. Accordingly, as anticipated, a clear correlation emerges: shorter Au–Ir distances at the PECs minima correspond to higher bond indices. Moreover, the results of this analysis are consistent with the interaction energy trend observed across the model systems in the PECs analysis, where the ionic models generally exhibit stronger interactions due to additional stabilization from Coulombic forces.

For a more detailed analysis of the Au­(I)···Ir­(I) interactions, we computed the recently proposed penetration index (*p*_AB_),^55^ which evaluates the extent of van der Waals shell overlap between atoms. Defined as the ratio of the intersecting region of two van der Waals crusts and the sum of their crust widths (eq S4), *p*_AB_ provides a qualitative measure of interaction strength: values near 0% reflect van der Waals contacts, while those above 100% suggest covalent or ionic bonding. The distribution of aurophilic interactions penetration indices ranges from 40 to 80%, with a maximum at 68%. For our model systems (see Table 2), the *p*_Au–Ir_ varied from 76.9 to 97.2%, slightly exceeding typical values reported for metallophilic interactions. These elevated *p*_Au–Ir_ values align with the interaction strength trends observed in the PECs and bond order analyses, supporting the presence of moderately strong noncovalent Au­(I)···Ir­(I) interactions that remain below the threshold characteristic of covalent bonding. For comparison, similar Pt···Pt and Pd···Pd interactions have been reported with penetration values exceeding 90%, often attributed to strong electrostatic attraction between charged species.^56^

To elucidate the physical origin of the interactions displayed in model systems, natural energy decomposition analysis (NEDA) was performed at the PBE0-D3­(BJ)/def2-TZVPP level of theory. To the best of our knowledge, this type of analysis is not implemented at the MP2 level of theory within Gaussian 16 software. Consequently, we employed a DFT approach incorporating the D3­(BJ) empirical dispersion correction, since PECs analysis shows that this method yields results comparable to those obtained with MP2. In this context, our analysis focuses on qualitative trends across model systems rather than on absolute interaction energies. The decomposition scheme partitions the total interaction energy into distinct physical components, including charge transfer, electrostatic contribution (such as polarization, Coulombic, and dipole–dipole contributions), and core repulsion (encompassing exchange-correlation effects, self-polarization, and Pauli repulsion). The most relevant energy components are depicted in Figure 6, while the complete data is provided in Table S10. Additional details regarding the NEDA methodology are available in the Supporting Information.

**Figure 6:**
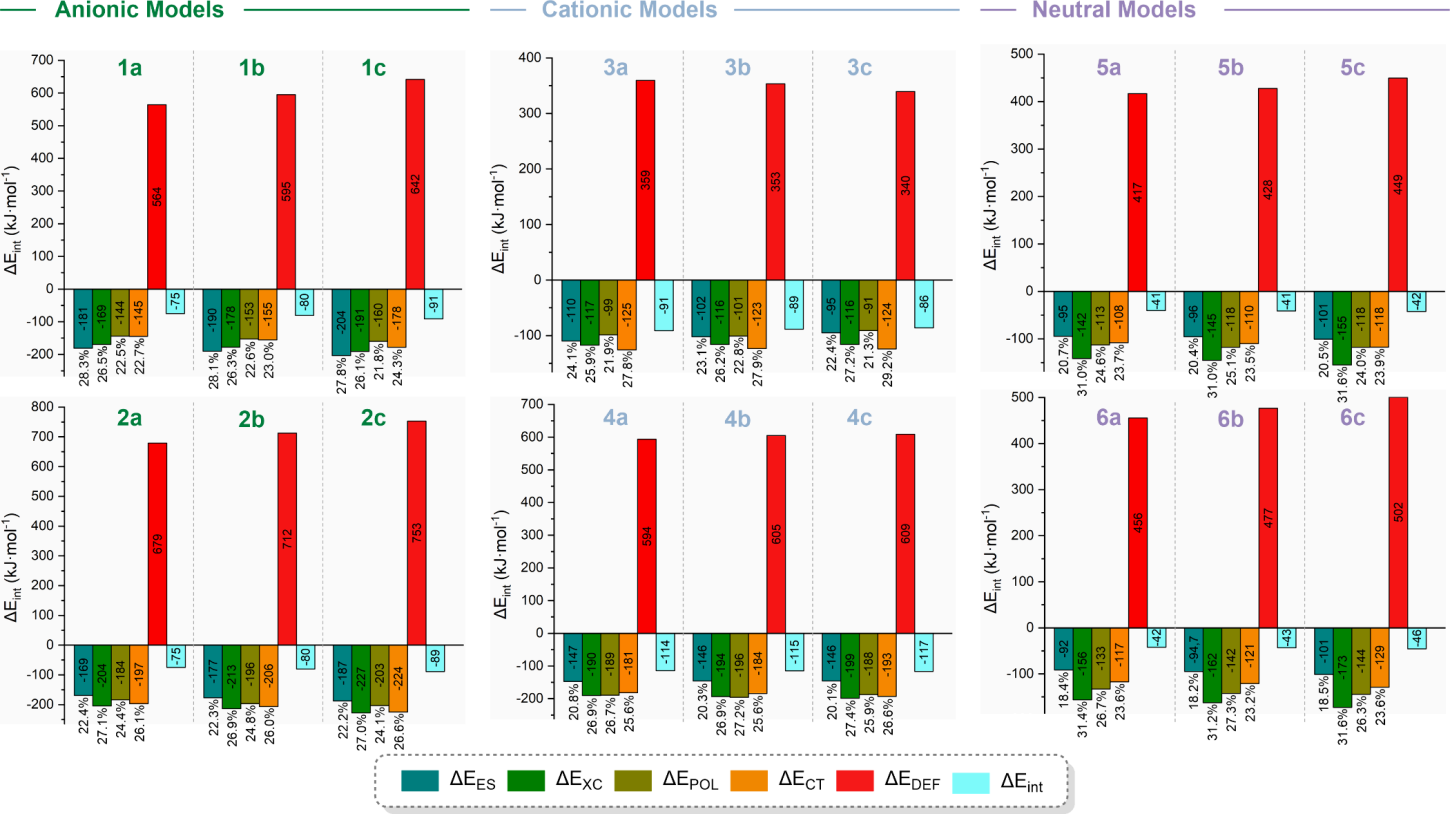
Natural energy decomposition analysis (NEDA) plots at the PBE0-D3­(BJ)/def2-TZVPP level of theory. Energy values are given in kJ mol^–1^ and the percentage contributions are calculated relative to the total stabilizing energy, as defined in eq S7. Raw data are provided in Table S10.

The total interaction energies ΔE_int_ are consistent with the trends established in previous analyses (i.e., **4** > **2** ≈ **1** > **3** > **5** ≈ **6**). As shown in the PECs analysis, the Δ*E*_int_ values computed at the PBE0-D3­(BJ) level of theory are consistently lower than those obtained with MP2, reflecting the different treatment of electron correlation. Consequently, these interaction energies are not directly comparable. In addition, the Δ*E*_int_ values obtained from both NEDA and PECs analyses at the PBE0-D3­(BJ) level of theory show good agreement.

The partitioning of molecular interactions reveals insightful details about the nature of the interactions. In anionic model systems **1**, the electrostatic (Δ*E*_ES_) and electron exchange and correlation effects (Δ*E*_XC_) are the main contributors, each accounting for approximately 27 to 28% of the total interaction energy. These are closely followed by charge transfer (Δ*E*_CT_) and polarization effects (Δ*E*_POL_), which contribute around 22 to 24%. In contrast, in model systems **2**, the dominant contributions are Δ*E*_XC_ (ca. 27%) and Δ*E*_CT_ (ca. 26%), followed by Δ*E*_POL_ (ca. 24%) and Δ*E*_ES_ (ca. 22%). Thus, the electrostatic component shifts from being the primary contributor in model systems **1** to a minor one in model systems **2**.

In cationic model systems **3**, the dominant contributions to total energy are Δ*E*_CT_ and Δ*E*_XC_ accounting for 28–29% and 26–27%, respectively. These are followed by Δ*E*_ES_ (22–24%) and Δ*E*_POL_ (ca. 21–23%). In the case of model systems **4**, Δ*E*_XC_, Δ*E*_POL_ and Δ*E*_CT_ contributions are all significant, accounting for 26 to 27% to the total interaction, while Δ*E*_ES_ decreases to about 20–21%. The key distinction between these two model systems lies in the increased contribution of polarization effects and decrease of the electrostatic component in model systems **4**. Consequently, the interaction strength rises with this increase, as model systems **4** exhibit the strongest interaction, while model systems **3** show the weakest among the series of ionic model systems.

In both neutral model systems **5** and **6**, as expected, the Δ*E*_ES_ contribution decreases to 18–20% approximately. The dominant term is Δ*E*_XC_, which reaches the highest values across all model systems at around 31%, followed by Δ*E*_POL_ contributing 25 to 27%, and Δ*E*_CT_ accounting for approximately 23%.

Overall, the energy components display comparable contributions, with no single term overwhelmingly dominating the interaction, comparing with Au­(I)···Pt­(II) interactions.^23f^ The dominant attractive contribution arises from the exchange-correlation (Δ_XC_) term, while the electrostatic component (Δ*E*_EL_) plays a role that is less significant than might be expected, emphasizing the dispersive nature of the interactions in the systems. Notably, as commonly observed in metallophilic systems, the Pauli repulsion (Δ*E*_DEF_) component is the largest one.^14a^ However, it is effectively counterbalanced and overcomes by the sum of the attractive interactions.

### 3.5 Topological Analysis of Electron Density

To further explore Au­(I)···Ir­(I) interactions, a combined QTAIM and IGMH analysis of the electron density was performed on the structures corresponding to the PECs minima, computed at the MP2/def2-TZVPP level of theory.

Within the framework of the quantum theory of atoms in molecules (QTAIM), a bond critical point (BCP) in the electron density is found between any pair of bonded atoms in a molecule.^48^ This BCP is accompanied by a bond path that represents the trajectory of the maximum electron density gradient connecting the corresponding nuclei. It is important to note that the physical interpretation of bond paths remains a topic of ongoing debate, as they do not exclusively represent classical Lewis-type bonds, but can also reflect weaker interactions such as van der Waals forces.^57^ The local topological properties evaluated at BCPs provide valuable insights into the nature and strength of bonding interactions, making this approach a widely used tool for characterizing a broad range of interatomic interactions.^9c,23d,58^ Accordingly, in all model systems, (3, −1) BCPs were detected along the bond path connecting the gold and iridium atoms, indicating the presence of interactions. The corresponding QTAIM descriptors associated with these BCPs are presented in Table 3.

**Table 3:** Descriptors of the QTAIM (3, −1) Bond Critical Points between Gold and Iridium Atom Calculated with the MP2/def2-TZVPP Electron Density^a^

**model**	***R***_**Au–Ir**_	**sign (λ**_**2**_ **)·ρ**_**e**_ **(*r*)**	**∇** ^**2**^ **ρ**_**e**_ **(*r*)**	***H*(*r*) × 10** ^**3**^	***G*(*r*)**	***V*(*r*)**	|*V*(*r*)|/*G*(*r*)	***G*(*r*)/ρ**_**e**_	***E***_**int**_ ^b^	***E***_**int**_ ^c^
**1a**	2.889	–0.044	+0.090	–8.948	+0.031	–0.0403	1.286	0.712	–52.9	–35.3
**1b**	2.864	–0.046	+0.093	–9.874	+0.033	–0.0430	1.298	0.719	–56.4	–37.3
**1c**	2.823	–0.049	+0.099	–11.485	+0.036	–0.0476	1.318	0.730	–62.4	–40.6
**2a**	2.892	–0.044	+0.087	–8.766	+0.031	–0.0392	1.288	0.700	–51.5	–34.3
**2b**	2.873	–0.045	+0.090	–9.474	+0.032	–0.0413	1.298	0.705	–54.2	–35.8
**2c**	2.840	–0.048	+0.094	–10.714	+0.034	–0.0449	1.314	0.713	–58.9	–38.5
**3a**	3.158	–0.028	+0.059	–3.216	+0.018	–0.0212	1.179	0.645	–27.8	–20.2
**3b**	3.183	–0.027	+0.057	–2.883	+0.017	–0.0199	1.169	0.638	–26.1	–19.2
**3c**	3.204	–0.026	+0.055	–2.642	+0.016	–0.0189	1.162	0.631	–24.9	–18.4
**4a**	2.850	–0.049	+0.094	–10.965	+0.034	–0.0453	1.319	0.702	–59.5	–38.7
**4b**	2.850	–0.049	+0.093	–10.987	+0.034	–0.0452	1.321	0.698	–59.3	–38.5
**4c**	2.845	–0.050	+0.093	–11.221	+0.034	–0.0456	1.327	0.694	–59.8	–38.7
**5a**	3.079	–0.032	+0.066	–4.303	+0.021	–0.0252	1.206	0.663	–33.1	–23.5
**5b**	3.068	–0.032	+0.068	–4.524	+0.021	–0.0259	1.212	0.0665	–34.0	–24.1
**5c**	3.026	–0.035	+0.072	–5.364	+0.023	–0.0287	1.230	0.675	–37.7	–26.3
**6a**	3.114	–0.029	+0.063	–3.588	+0.019	–0.0229	1.185	0.658	–30.1	–21.8
**6b**	3.114	–0.029	+0.063	–3.602	+0.019	–0.0230	1.186	0.658	–30.2	–21.8
**6c**	3.108	–0.030	+0.064	–3.705	+0.020	–0.0234	1.189	0.659	–30.7	–22.1

a M–M distance (*R* in Å), product of the sign of second largest eigenvalue of Hessian matrix of electron density (sign­(λ_2_)·ρ_e_(*r*) in a.u.), Laplacian of electron density (∇^2^ρ_e_(*r*) in a.u.), electron energy density (*H*(*r*) in a.u.), Lagrangian kinetic energy density (*G*(*r*) in a.u.), potential energy density (*V*(*r*) in a.u.), ratio |*V*(*r*)|/*G*(*r*), ratio |*V*(*r*)|/2*G*(*r*), ratio *G*(*r*)|/ρ_e_ in a.u., estimated energies (*E*_int_ in kJ mol^–1^).

b *E*_int_ = −*V*(*r*)/2.

c *E*_int_ = 0.429*G*(*r*).

Thus, the analysis of QTAIM descriptors provides insight into the nature and strength of BCPs. High electron density (ρ_e_(*r*) > 0.2 a.u.) with negative ∇^2^ρ_e_(*r*) and *H*(*r*) values indicates shared-shell (covalent) interactions, while low ρ_e_(*r*) less than 0.1 a.u. with positive ∇^2^ρ_e_(*r*) and *H*(*r*) values reflects closed-shell (noncovalent) interactions, such as ionic or van der Waals forces.^59a^ Thus, the opposite signs of ∇^2^ρ_e_(*r*) and *H*(*r*) in Table 3 indicate that the Au­(I)···Ir­(I) interactions are of intermediate character, combining features of both ionic and covalent bonding with partial electron sharing. The low ρ_e_(*r*) values at the BCPs support their classification as noncovalent, closed-shell interactions with donor–acceptor character, while the negative sign of ρ_e_(*r*) denotes their attractive nature.^59b^ Additionally, the interaction strength follows the trend **4** < **1** ≈ **2** < **5** ≈ **6** < **3** across model systems, in good agreement with previous analyses. Although the halide variation generally increases the interaction strength from Cl to I, the differences remain minimal.

A more precise classification of the interactions can be achieved by analyzing the Lagrangian kinetic energy density, *G*(*r*), together with the potential energy density, *V*(*r*). When *V*(*r*) dominates locally, it signifies a charge concentration typical of shared-shell (covalent) interactions; conversely, dominance of the *G*(*r*) indicates charge depletion associated with closed-shell interactions. The ratio |*V*(*r*)|/*G*(*r*) categorizes closed-shell interactions by covalency degree: values below 1 correspond to pure closed-shell (noncovalent) interactions; values above 2 indicate covalent electron sharing; and intermediate values from 1 to 2 reflect partial covalent character, known as regular closed-shell interactions (regular CS).^60^ The |*V*(*r*)|/*G*(*r*) ratios listed in Table 3 range from approximately 1.15 to 1.35, indicating a regular CS type interaction, consistent with the previous QTAIM descriptors analysis. Furthermore, the increase in this ratio correlates with stronger Au­(I)···Ir­(I) interactions, reflecting a higher degree of covalency in the model systems.

Based on the *G*(*r*)/ρ_e_ ratio, closed-shell interactions are classified as metallic (shared) bonding when *G*(*r*)/ρ_e_ < 1, and as donor–acceptor interactions when the ratio is approximately 1.^61^ The results range from approximately 0.66 to 0.71 (Table 3), which are typical for metallic shared bonding. Likewise, model systems with stronger Au­(I)···Ir­(I) interactions, such as **4, 1**, and **2**, exhibit higher values, indicating an increased donor–acceptor character, which correlates with a similar trend in covalency.

To conclude, we estimated the metallophilic interaction energies using the methods proposed by Espinosa and Vener.^62^ The computed Δ*E*_int_ values range from 25 to 62 kJ mol^–1^ (Espinosa) and from 18 to 40 kJ mol^–1^ (Vener). The halogen substitution results in only a marginal increase in the interaction energy (about 1 kJ mol^–1^). Furthermore, these energy values qualitatively correlate with the trends observed in the ρ_e_(*r*) at the BCPs and PECs analysis.

In summary, the QTAIM analysis (i) identifies a BCP and associated bond path between gold and iridium atoms in all model systems, (ii) reveals descriptors consistent with a regular CS interaction exhibiting partial electron sharing and characteristics of metallic bonding (iii) indicates Δ*E*_int_ within the range of noncovalent and dispersive forces; and (iv) shows that the ρ_e_ and Δ*E*_int_ values for the Au­(I)···Ir­(I) interactions are comparable to, or even exceed, those typically observed for Au­(I)···Au­(I) contacts,^58a^ and are significantly higher than those reported for Au­(III)···Au­(III) and Au­(I)···Pt­(II) interactions,^23e,23f^ highlighting the relatively stronger nature of the Au­(I)···Ir­(I) interactions.

To visualize the interactions bearing in model systems, the independent gradient model based on Hirshfeld partition of molecular electron density (IGMH) was computed.^49^ This recent real-space method employs the reduced density gradient (RDG) to effectively visualize and analyze both covalent and noncovalent interactions. Compared to the widely used noncovalent interaction (NCI) method, IGMH offers notable advantages, such as quantitative interaction indices and clearer, more interpretable isosurfaces.^63^

The RDG identifies interaction regions: low RDG and low electron density suggest weak interactions, while low RDG with high density indicates stronger ones. The sign of the second largest eigenvalue (λ_2_) of the Hessian of the electron density distinguishes their nature; negative λ_2_ indicates attraction, and positive λ_2_, repulsion. These features are visualized through isosurfaces weighted by sign­(λ_2_)·ρ_e_(*r*) and colored according to a Blue-Green-Red (BGR) scale: blue to green indicates attractive interactions (sign­(λ_2_)·ρ_e_(*r*) < 0), green corresponds to van der Waals interactions (sign­(λ_2_)·ρ_e_(*r*) ≈ 0), and green to red reflects repulsive interactions (sign­(λ_2_)·ρ_e_(*r*) > 0). Accordingly, Figure 7 displays the IGMH isosurfaces along with the BCPs and bond paths obtained from the QTAIM and IGMH analyses.

**Figure 7:**
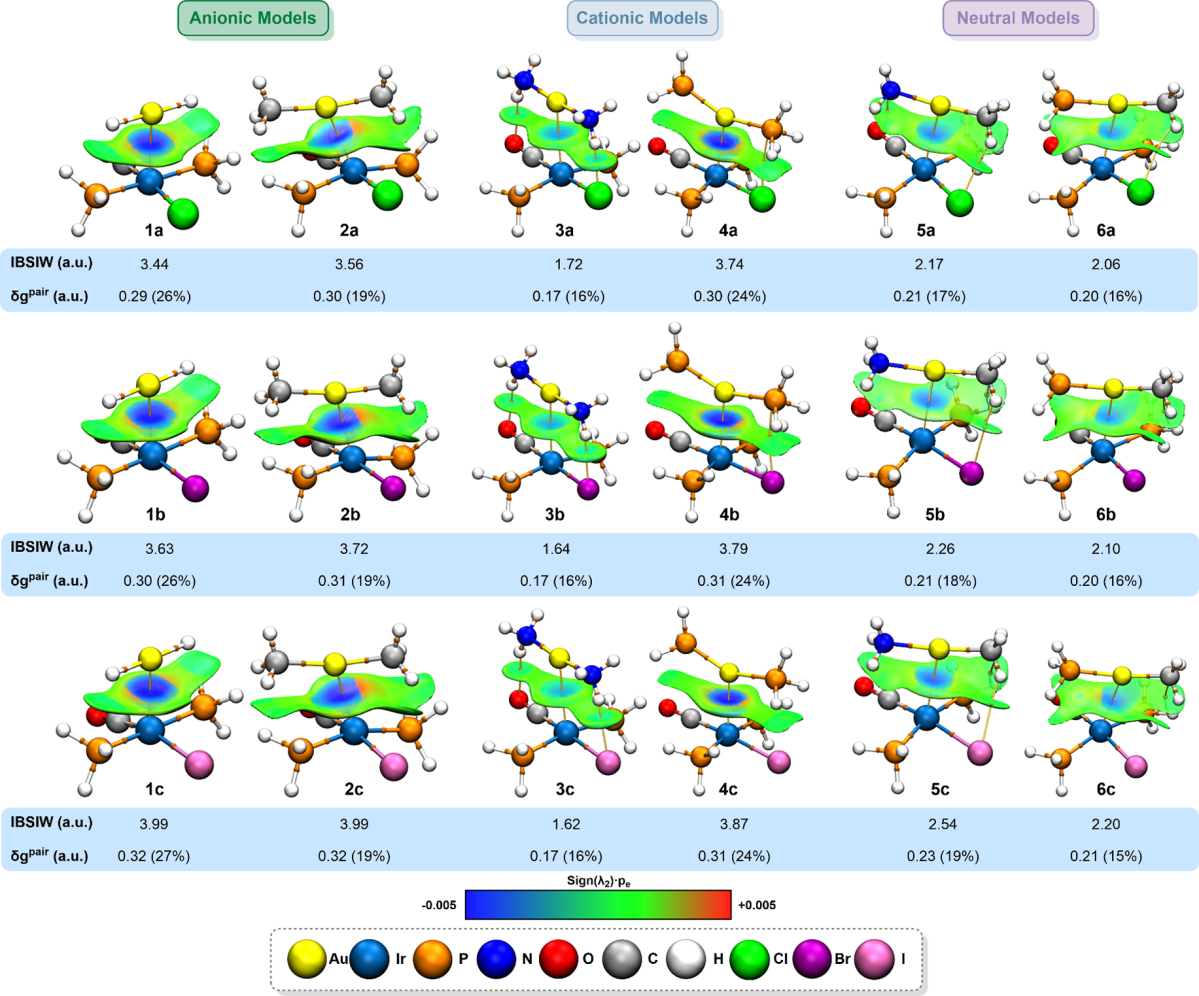
QTAIM (3, −1) BCPs (orange dots), bond paths (yellow strings), and the IGMH isosurface (isovalue δg^inter^ = 0.005 a.u.) representations of models **1**–**6**.

In all model systems, a bluish region was observed between the metal centers, corresponding to an electron-rich area (ρ_e_(*r*) > 0) with an attractive character (λ_2_ < 0). Together with the presence of bond critical points (BCPs) and bond paths, it provides definitive topological evidence for an attractive interaction between the gold and iridium atoms. The surrounding green regions correspond to typical van der Waals interactions involving the ligands. Additionally, several secondary BCPs and bond paths were identified. Specifically, model systems **3** feature N–H···X and N–H···O=C contacts, while model systems **4** present P–H···X interactions. In model systems **5**, both C–H···X and P–H···C interactions were found, and model systems **6** exhibit additional P–H···C contacts.

To quantify the Au­(I)···Ir­(I) interactions, we analyzed some IGMH indices. The atomic pair δg index (δG^pair^) assesses the contribution of specific atom pairs to the interaction, while the atomic δg index (δG^atom^) evaluates the individual role of each atom. Additionally, the intrinsic bond strength index for weak interactions (IBSIW) enables differentiation of interaction strengths. In all cases, larger index values indicate stronger interactions. The δG^atom^ and IBSIW indices are illustrated in Figure 7, while the δG^atom^ values are provided in Tables S11–16. The δG^pair^ and IBSIW values confirm the strength order, as in previous analyses. Evaluation of these quantitative indices for additional interactions (Tables S17–S20) reveals that metallophilic contacts are the strongest and thus represent the primary stabilizing interactions. These are further supported by secondary interactions, such as hydrogen and halogen bonding. Moreover, the δG^atom^ results further indicate that the gold center plays a more prominent role in the stabilization of the interactions, contributing approximately 39 to 65%, whereas the iridium atom accounts for only 33 to 37%.

## 4 Conclusions

Unsupported model systems inspired by previously reported experimental Au–Ir heterobimetallic complexes were investigated through quantum chemical and topological analyses with particular emphasis on Au­(I)···Ir­(I) interactions. Computed Au–Ir distances and interaction energies at post Hartree–Fock levels of theory support the existence of metallophilic interactions across all of the proposed model systems.

The Au­(I)···Ir­(I) interactions are classified as regular closed-shell interactions, exhibiting partial electron-sharing and features characteristic of metallic bonding. Their strength, ranging from approximately 20 to 60 kJ mol^–1^, is comparable to, or even exceeds, that of the well-known Au­(I)···Au­(I) interactions (ca. 20–50 kJ mol^–1^) and clearly exceeds those reported for Au­(I)···Pt­(II) or Au­(III)···Au­(III) interactions (ca. 15 kJ mol^–1^). The strongest metallophilic interactions are found in the cationic model systems **4**, which contain the [Au­(PH_3_)_2_]^+^ fragment, followed by the anionic model systems **2** and **1**, featuring [Au­(CH_3_)_2_]^−^ and [AuH_2_]^−^ fragments, respectively. Additionally, substitution of the halide ligand on the iridium center leads to a moderate increase in interaction energy, following the trend Cl < Br < I. Relativistic effects were found to modulate metallophilic interactions (2–15%), albeit less significantly than in model systems bearing Au­(I)···Au­(I) or Au­(I)···Hg­(II) interactions.

We demonstrated that electron correlation effects are essential for accurately describing these types of interactions. Accordingly, NBO, NEDA, and bond order calculations confirm that Au­(I)···Ir­(I) interactions are noncovalent and predominantly dispersive in nature. While other noncovalent forces such as hydrogen and halogen bonding contribute to overall system stability, Au­(I)···Ir­(I) interactions prevail as the dominant force. Topological analyses of the electron density of QTAIM and IGMH consistently reveal a bond critical point (BCP) along the Au­(I)···Ir­(I) bond path in all model systems, providing definitive evidence of an attractive interaction between these metals.

This study underscores the strong qualitative consistency among various quantum chemical and topological electron density approaches for the characterization of metallophilic interactions. Computational insights not only reinforce the reliability of these methodologies but also provide a valuable framework for guiding future synthetic strategies. These findings emphasize the relevance of such noncovalent-type interactions in the rational design of unsupported gold–iridium heterobimetallic complexes, and follow the path laid out by our recent studies,^23e,23f^ further consolidating the role of quantum chemical calculations in the understanding and design of metallophilic systems.

## Supplementary Material



## Notes

Computational details, optimized structures, potential energy curves calculations, NBO effective charges, NEDA, IGMH analysis, and Cartesian coordinates of the optimized geometries for model systems **1–6** (PDF)

## Abbreviations

BCPs: bond critical points
BSSE: basis set superposition error
CS: closed-shell
D3BJ: Grimme′s third-generation dispersion correction with Becke-Johnson damping
DE: delocalization error
def2-TZVPP: Karlsruhe basis sets: triple-ζ valence with doble polarization functions
DFT: density functional theory
DPLNO–CCSD­(T): domain-based local pair natural orbital coupled cluster with single, double, and perturbative triple excitations
ECPs: effective core potentials
FBO: fuzzy bond order
IBSI: intrinsic bond strength index
IBSIW: intrinsic bond strength index for weak interactions
IGMH: independent gradient model based on Hirshfeld partition analysis
MP2: second-order Mo̷ller-Plesset perturbation theory
NBO: natural bond orbital
NCI: noncovalent interactions
NEDA: natural energy descomposition analysis
PBE0: Perdew–Burke–Ernzerhof hybrid functional
PECs: potential energy curves
QTAIM: quantum theory of atoms in molecules
RDG: reduced density gradient
RHF: restricted Hartree–Fock theory
RI: resolution of identity approximation
RMSD: root-mean-square deviation
SCS-MP2: spin-component-scaled variant of Second-order Mo̷ller-Plesset perturbation theory
WBO: Wiberg bond order
ZORA: zero-order regular approximation
