# Supplementary material for: Computational Characterization of Unsupported Au(I)···Ir(I) Metallophilic Interactions: Evidence for Strong Dispersive Stabilization
Source: Inorg Chem. 2025 Aug 18;64(34):17569–84. doi: 10.1021/acs.inorgchem.5c03295 (PMC12406185; doi:10.1021/acs.inorgchem.5c03295)
Supplement: Supplementary file 1 [file ic5c03295_si_001.pdf]

Computational Characterization of Unsupported Au(I)···Ir(I) Metallophilic Interactions: Evidence for Strong Dispersive Stabilization

Félix Reboiro, M. Elena Olmos, José M. López-de-Luzuriaga* and Miguel Monge*

Departamento de Química, Instituto de Investigación en Química de la Universidad de La Rioja (IQUR), Madre de Dios 53, 26006 Logroño, La Rioja, Spain.

E-mail: josemaria.lopez@unirioja.es, miguel.monge@unirioja.es

Supporting Information

Table of Contents

Computational Details S1

Optimized Structures S5

Potential Energy Curves (PECs) S7

NBO Effective Charges S13

NEDA S14

IGMH Analysis S15

Cartesian Coordinates S18

Computational Details

All model systems were fully optimized using the Gaussian 16 Revision C.01 suite of programs[1] at the MP2[2,3] and RHF[4] levels of theory with def2-TZVPP basis sets[5] for all atoms, and 60 electron effective core potentials for gold and iridium atoms (def2-ECPs).[6] The frequency analyses at the same level of theory were carried out to verify that the optimized geometries belong to minima (no imaginary frequencies were found). The model systems were built from scratch and fully optimized without geometrical constraints. The structures were visualized and rendered using GaussView 6.1[7] and UCSF ChimeraX 1.3 visualization programs.[8]

The relativistic effects of model systems **a** were studied using the zero order regular approximation (ZORA).[9] The model systems were fully optimized at the SCS-MP2-ZORA level of theory,[10] using the SARC-ZORA-TZVPP basis sets for the metal atoms and ZORA-SVP for the remaining atoms.[11] Nonrelativistic ZORA calculations were carried out by setting the ZORA method with the inverse fine structure constant to 100*c*, referring *c* to the speed of light in vacuum. Therefore, these levels of theory are abbreviated as SCS-MP2-ZORA for standard ZORA calculations and SCS-MP2-ZORA^{NR} for nonrelativistic ZORA calculations. These calculations were performed with ORCA program (version 5.0.4).[12] The RIJCOSX[13] approximation was applied to standard ZORA calculations. Numerical frequency analysis of the optimized geometries was conducted to confirm that they belong to minima (no imaginary frequencies were found). Interaction energies were calculated using the counterpoise (cp) correction at their respective levels of theory, employing the SARC-ZORA-TZVPP basis sets for the metal atoms and ZORA-TZVPP for all other atoms. Structural alignment was performed by superimposing the iridium atoms, followed by RMSD calculation using the VMD 1.9.4a53 visualization program package.[14]

Interaction energies (ΔE_{int}) were obtained at the MP2, RHF and PBE0-D3BJ[15] levels of theory with the def2-TZVPP basis sets using eq (**S1**). Therefore, a counterpoise correction to the basis set superposition error (BSSE) on interaction energies was performed,[16]

$$\Delta E_{int} = E_{AB}^{(AB)} - E_{A}^{(AB)} - E_{B}^{(AB)} \qquad \textbf{\textit{S1}}$$

where ΔE_{int} represents the interaction energy, $E_{AB}^{(AB)}$ is the energy of the dimer and $E_{A}^{(AB)}$, $E_{B}^{(AB)}$ are monomer´s energies calculated using the basis sets of the dimer.

The calculated points were fitted using a four-parameter (see eq (**S2**)), which had been previously used to derive the Herschbach-Laurie function,[17]

$$\Delta E_{int}\,(R) = Ae^{-BR} - C \cdot R^{D} \qquad \textbf{\textit{S2}}$$

where R is the corresponding interatomic distance and A, B, C, D are fitting parameters. The internal coordinate corresponding to the interatomic distance was manually stretched to the selected values whereas all other internal coordinates were kept intact.

The electron correlation contribution to the total interaction energy at the minima of the potential energy curves (PECs) was calculated by subtracting the interaction energy at the MP2 level of theory at the MP2 minimum distance ($\Delta E_{\min MP2}^{MP2}$) from the interaction energy at the RHF level of theory at the MP2 minimum distance ($\Delta E_{\min MP2}^{RHF}$) and dividing it by the first term, as shown in eq (**S3**). Single point calculations of these points at MP2/def2-TZVP in the RHF and MP2 minima of the PECs were performed to refine the energies values. Additional single-point counterpoise-corrected energy calculations at the SCS-MP2 and DLPNO-CCSD(T)[18] levels of theory using the def2-TZVPP basis sets and the RIJCOSX approximation were performed in the ORCA 5 software package.

$$electron\ correlation\ contribution = \frac{\Delta E_{\min MP2}^{MP2} - \Delta E_{\min MP2}^{RHF}}{\Delta E_{\min MP2}^{MP2}} \cdot 100 \qquad \textbf{\textit{S3}}$$

NBO analysis[19] and Wiberg Bond Order (WBO)[20] calculations were carried out in each model system using Gaussian 16, with the aim of computing the natural effective charges within the metal centers and their bonded atoms. Additionally, the intrinsic bond strength index (IBSI)[21] and the fuzzy bond order[22] were calculated at the MP2/def2-TZVPP level of theory using the Multiwfn 3.8 software package.[23]

The penetration index (p_{AB}), recently proposed by Santiago Álvarez *et al.*,[24] was calculated using eq (**S4**),

$$p_{AB}(\%) = \frac{(v_A + v_B - d_{AB})}{(v_A + v_B - r_A - r_B)} \cdot 100 \qquad \textbf{\textit{S4}}$$

where v is the corresponding van der Waals radii, r the corresponding covalent radii and d the interatomic distance in the computational model systems. Additionally, the covalent and van der Waals radii proposed by the same authors were employed.[25,26]

Natural energy decomposition analysis (NEDA)[27,28] was carried out for all model systems using Gaussian 16 and NBO 7.0 programs.[29] Calculations were performed at the DFT[30,31] Perdew–Burke-Ernzerhof (PBE0)[14] hybrid functional using the third empirical dispersion correction by Grimme D3(BJ).[32,33] It allows for the splitting of the interaction energy between monomers into electrical interaction (E_{El}), charge transfer (E_{CT}) and core repulsion (E_{CORE}) components (eq (**S5**)).

$$E_{int} = E_{CT} + E_{CORE} + E_{EL} \qquad \textbf{\textit{S5}}$$

The core repulsion component (E_{CORE}) encompasses the sum of the Pauli repulsion (E_{DEF}), electron exchange and correlation effects (E_{EX}) minus the self-polarization energy (E_{ES}) of the components (eq (**S6**)).

$$E_{CORE} = E_{DEF} + E_{XC} - E_{SE} \qquad \textbf{\textit{S6}}$$

The electrical interaction (E_{EL}) is represented by the sum of the electrostatic components (E_{ES}), the sum of the polarization effects (E_{POL}) and the self-polarization (E_{ES}) energy of the components, which the latter is destabilizing in nature. The percentages of the stabilizing components were calculated by dividing the corresponding attractive component by the sum of all the others (eq (**S7**)).

$$NEDA\ percentage = \frac{|E_{CT}|\ or\ |E_{ES}|\ or\ |E_{POL}|\ or\ |E_{XC}|}{|E_{CT} + E_{ES} + E_{POL} + E_{XC}|} \cdot 100 \qquad \textbf{\textit{S7}}$$

An in-depth topological analysis on each computational model system was performed for the purpose of analyzing the interaction nature from a qualitative point of view. The topology and properties of the MP2/def2-TZVPP electron density of the structures have been examined using quantum theory of atoms in molecules (QTAIM)[34] and independent gradient model based on Hirsfeld partition (IGMH)[35] methods using Multiwfn 3.8 software. Additionally, VMD 1.9.4a53 visualization program package was employed for the representations of the electron density studied in each analysis.

References

1. M. J. Frisch, G. W. Trucks, H. B. Schlegel, G. E. Scuseria, M. A. Robb, J. R. Cheeseman, G. Scalmani, V. Barone, G. A. Petersson, H. Nakatsuji, X. Li, M. Caricato, A. V. Marenich, J. Bloino, B. G. Janesko, R. Gomperts, B. Mennucci, H. P. Hratchian, J. V. Ortiz, A. F. Izmaylov, J. L. Sonnenberg, D. Williams-Young, F. Ding, F. Lipparini, F. Egidi, J. Goings, B. Peng, A. Petrone, T. Henderson, D. Ranasinghe, V. G. Zakrzewski, J. Gao, N. Rega, G. Zheng, W. Liang, M. Hada, M. Ehara, K. Toyota, R. Fukuda, J. Hasegawa, M. Ishida, T. Nakajima, Y. Honda, O. Kitao, H. Nakai, T. Vreven, K. Throssell, J. A. Montgomery, Jr., J. E. Peralta, F. Ogliaro, M. J. Bearpark, J. J. Heyd, E. N. Brothers, K. N. Kudin, V. N. Staroverov, T. A. Keith, R. Kobayashi, J. Normand, K. Raghavachari, A. P. Rendell, J. C. Burant, S. S. Iyengar, J. Tomasi, M. Cossi, J. M. Millam, M. Klene, C. Adamo, R. Cammi, J. W. Ochterski, R. L. Martin, K. Morokuma, O. Farkas, J. B. Foresman and D. J. Fox, *Gaussian 16 Rev. C.01*, Wallingford CT, 2016.
2. C. Møller and M. S. Plesset, *Phys. Rev.,* 1934, **46**, 618.
3. M. J.Frisch and M. Head-Gordon.; J. A. Pople, *Chem. Phys. Lett.*, 1990, 166, 275.
4. C. C. J. Roothaan, *Rev. Mod. Phys.*, 1951, **23**, 69.
5. F. Weigend and R. Ahlrichs, *Phys. Chem. Chem. Phys.,* 2005, **7**, 3297.
6. D. Andrae, U. Häußermann, M. Dolg, H. Stoll and H. Preuß, *Theor. Chim. Acta* 1990, **77**, 123.
7. R. Dennington, T. A. Keith and J. M. Millam, *GaussView Version 6*, Semichem Inc., Shawnee Mission, KS, **2016**.
8. E. F. Pettersen, T. D. Goddard, C. C. Huang, E. C. Meng, G. S. Couch, T. I. Croll, J. H. Morris and T. E. Ferrin, *Protein Sci.,* 2020, **30**, 70.
9. E. van Lenthe, J. G. Snijders and E. J. Baerends, *J. Chem. Phys.*, 1996, **105**, 6505.
10. M. Gerenkamp and S. Grimme, *Chem. Phys. Lett.*, 2004, **392**, 229.
11. M. Bühl, C. Reimann, D. A. Pantazis, T. Bredow and F. Neese, *J. Chem. Theory Comput.*, 2008, **4**, 1449.
12. F. Neese, *WIREs Comput. Mol. Sci.*, 2022, **12**, e1606.
13. B. Helmich-Paris, B. de Souza, F. Neese, R. Izsák, *J. Chem. Phys.* 2021, **155**, 104109.
14. W. Humphrey, A. Dalke and K. Schulten, *J. Mol. Graph*., 1996, **14**, 3.
15. M. Ernzerhof and G. E. Scuseria, *J. Chem. Phys.*, 1999, **110**, 5029.
16. S. F. Boys and F. Bernardi, *Mol. Phys.*, 1970, **19**, 553.
17. D. R. Herschbach and V. W. Laurie, *J. Chem. Phys.,* 1961, **35**, 458.
18. C. Riplinger and F. Neese*, J. Chem. Phys.,* 2013, **138**, 034106.
19. E. D. Glendening, C. R. Landis and F. Weinhold, *WIREs Comput. Mol. Sci.*, 2011, **2**, 1.
20. K. B. Wiberg, *Tetrahedron,* 1968, **24**, 1083.
21. J. Klein, H. Khartabil, J.-C. Boisson, J. Contreras-García, J.-P. Piquemal and E. Hénon, *J. Phys. Chem. A*, 2020, 124, 1850.
22. I. Mayer and P. Salvador, *Chem. Phys. Lett*., 2004, **383**, 368.
23. T. Lu and F. Chen, *J. Comput. Chem.*, 2011, 33, 580
24. J. Echeverría and S. Alvarez, *Chem. Sci.*, 2023, **14**, 11647.
25. B. Cordero, V. Gómez, A. E. Platero-Prats, M. Revés, J. Echeverría, E. Cremades, F. Barragán and S. Alvarez, *Dalton Trans.*, 2008, 2832.
26. S. Alvarez, *Dalton Trans.*, 2013, **42**, 8617.
27. E. D. Glendening and A. Streitwieser, *J. Chem. Phys.*, 1994, **100**, 2900.
28. E. D. Glendening and *J. Phys. Chem. A,* 2005, **109**, 11936.

29. E. D. Glendening, J, K. Badenhoop, A. E. Reed, J. E. Carpenter, J. A. Bohmann, C. M. Morales, P. Karafiloglou and C. R. Landis, F. Weinhold, *NBO 7.0.*, Theoretical Chemistry Institute, University of Wisconsin, Madison, 2018.

30. P. Hohenberg and W. Kohn, *Phys. Rev.,* 1964, **136**, B864.

31. W. Kohn and L. J. Sham, *Phys. Rev.*, 1965, **140**, A1133.

32. S. Grimme, *J. Comput. Chem.,* 2006, **27**, 1787.

33. S. Grimme, J. Antony, S. Ehrlich and H. Krieg, *J. Chem. Phys.*, 2010, **132**, 154104.

34. R. F. W. Bader, *Chem. Rev.*, 1991, **91**, 893.

35. T. Lu and Q. Chen, *J. Comput. Chem*., 2022, **43**, 539.

Optimized Structures

Table S1 Selected bond lengths (Å) and bond angles (°) for structures optimized at the MP2 and RHF levels of theory.

Model	MP2/def2-TZVPP					RHF/def2-TZVPP						
	$D_{Au\text{-}Ir}$	$D_{Au\text{-}X}$	$A_{P\text{-}Ir\text{-}Au}$	$A_{L\text{-}Au\text{-}L}$[a]	$A_{X\text{-}Ir\text{-}CO}$[a]	$D_{Au\text{-}H\text{-}P}$	$D_{L\text{-}P\text{-}Ir}$	$D_{L\text{-}H\text{-}X}$	$D_{Au\text{-}X}$	$A_{P\text{-}Ir\text{-}Au}$	$A_{L\text{-}Au\text{-}L}$[a]	$A_{X\text{-}Ir\text{-}CO}$[a]
**1a**	2.8165	3.7118	87.12	178.01	171.17	3.2130	3.3327	-	-	157.03	179.56	178.83
**1b**	2.7955	3.8017	87.51	178.17	168.44	3.1836	3.3284	-	-	155.70	179.52	178.83
**1c**	2.7616	3.8955	87.91	178.37	162.92	3.1514	3.3226	-	-	154.20	179.48	178.90
**2a**	2.8237	3.8960	116.92	170.79	172.49	3.0144	4.0224	-	-	151.05	179.91	178.80
**2b**	2.8071	3.9854	117.04	170.30	170.93	3.0141	4.0224	-	-	151.02	179.91	178.80
**2c**	2.7812	4.0790	117.16	169.55	168.29	2.9415	3.9920	-	-	142.76	179.88	178.81
**3a**	3.0780	4.0362	90.80	174.16	178.74	-	-	2.3705	-	72.42	178.90	179.90
**3b**	3.0968	4.2129	94.73	173.65	179.85	-	-	2.5935	-	72.60	178.67	178.85
**3c**	3.1147	4.4567	94.58	173.03	178.46	-	-	2.9280	-	72.84	178.35	179.80
**4a**	2.8033	3.8113	93.58	155.56	178.64	-	-	-	3.4793	87.29	179.53	179.97
**4b**	2.8025	3.9810	93.50	153.90	177.40	-	-	-	3.5742	87.60	178.85	179.96
**4c**	2.7976	4.2337	93.30	151.93	175.25	-	-	-	3.7004	88.14	177.89	180.00
**5a**	2.9803	3.9755	110.51	174.57	175.38	3.4436	4.6702	-	-	105.30	179.51	179.66
**5b**	2.9645	4.0913	110.00	174.29	174.21	3.4336	4.6495	-	-	105.88	179.53	179.61
**5c**	2.9271	4.2436	108.54	174.03	172.06	3.4336	4.6500	-	-	105.88	179.53	179.61
**6a**	3.0263	3.7102	108.36	175.57	173.85	3.5799	4.6141	-	-	136.07	179.64	179.70
**6b**	2.9971	3.8577	113.87	172.73	173.77	3.5779	4.6093	-	-	135.17	179.62	179.68
**6c**	2.9971	3.8577	113.87	172.74	173.77	3.5722	4.6015	-	-	133.83	179.60	179.67

Distances in Å and angles in °. [a] The angles in the corresponding optimized fragment structures are 180°.

Table S2 Selected bond lengths (Å) and bond angles (°) for structures optimized at the RI-SCS-MP2-ZORA and SCS-MP2-ZORA^{NR} levels of theory.

Model	**RI-SCS-MP2-ZORA/SARC-TZVPP(M)/SVP**					**SCS-MP2-ZORA^{NR}/SARC-TZVPP(M)/SVP**				
	$D_{Au\cdots Ir}$	$D_{Au\cdots X}$	$A_{P\text{-}Ir\text{-}Au}$	$A_{L\text{-}Au\text{-}L}$	$A_{X\text{-}Ir\text{-}CO}$	$D_{Au\cdots Ir}$	$D_{Au\cdots X}$	$A_{P\text{-}Ir\text{-}Au}$	$A_{L\text{-}Au\text{-}L}$	$A_{X\text{-}Ir\text{-}CO}$
**1a**	2.938	3.914	86.58	176.70	174.13	3.080	4.293	86.67	178.68	176.43
**2a**	2.978	4.082	104.81	173.05	174.29	3.078	4.160	98.08	173.43	176.72
**3a**	3.153	4.103	96.64	173.77	178.20	2.953	3.907	92.79	152.09	177.15
**4a**	2.843	3.885	93.59	156.88	178.22	3.068	3.692	92.19	142.59	177.02
**5a**	3.1550	4.1340	106.2	173.4	177.3	3.1790	4.3810	102.20	171.20	176.40
**6a**	3.1490	3.7330	110.4	174.1	175.5	3.1890	3.665	113.10	169.40	178.70

Potential Energy Curves (PECs)

Table S3 Absolute energies (kJ·mol^{-1}) as function of Au^{I}-Ir^{I} distance (Å) of model systems **1** calculated at the RHF/def2-TZVPP, MP2/def2-TZVPP and PBE0-D3BJ/def2-TZVPP levels of theory with the counterpoise correction (cp) for the basis set superposition error (BSSE).

R	1a			1b			1c		
	ΔE_{int}^{RHF}	ΔE_{int}^{MP2}	ΔE_{int}^{PBE0}	ΔE_{int}^{RHF}	ΔE_{int}^{MP2}	ΔE_{int}^{PBE0}	ΔE_{int}^{RHF}	ΔE_{int}^{MP2}	ΔE_{int}^{PBE0}
2.4	173.11	12.26	41.05	161.20	-0.38	29.62	140.28	-22.26	9.20
2.6	64.54	-61.80	-40.63	55.41	-71.84	-49.54	39.32	-89.41	-65.73
2.8	12.79	-83.72	-70.33	5.83	-91.67	-77.32	-6.48	-105.60	-90.12
3.0	-11.27	-83.35	-76.36	-16.60	-89.54	-81.84	-26.00	-100.42	-91.96
3.2	-21.79	-74.77	-72.30	-25.90	-79.54	-76.61	-33.12	-87.99	-84.56
3.4	-25.64	-64.14	-64.48	-28.86	-67.82	-67.86	-34.47	-74.31	-74.18
3.6	-26.21	-53.89	-55.86	-28.78	-56.74	-58.53	-33.20	-61.71	-63.51
3.8	-25.20	-44.81	-47.66	-27.29	-47.03	-49.83	-30.84	-50.88	-53.81
4.0	-23.48	-37.11	-40.42	-25.21	-38.83	-42.13	-28.12	-41.84	-45.31

Table S4 Absolute energies (kJ·mol^{-1}) as function of Au^{I}-Ir^{I} distance (Å) of model systems **2** calculated at the RHF/def2-TZVPP, MP2/def2-TZVPP and PBE0-D3BJ/def2-TZVPP levels of theory with the counterpoise correction (cp) for the basis set superposition error (BSSE).

R	2a			2b			2c		
	ΔE_{int}^{RHF}	ΔE_{int}^{MP2}	ΔE_{int}^{PBE0}	ΔE_{int}^{RHF}	ΔE_{int}^{MP2}	ΔE_{int}^{PBE0}	ΔE_{int}^{RHF}	ΔE_{int}^{MP2}	ΔE_{int}^{PBE0}
2.4	235.32	22.68	62.34	224.47	11.05	51.76	206.38	-7.82	34.35
2.6	103.58	-63.18	-33.47	94.95	-72.72	-42.01	80.93	-87.82	-55.73
2.8	39.49	-88.99	-69.12	32.75	-96.73	-75.98	22.06	-108.70	-86.78
3.0	8.67	-88.78	-77.15	3.45	-94.98	-82.63	-4.61	-104.31	-91.13
3.2	-5.79	-78.95	-73.35	-9.84	-83.85	-77.70	-15.90	-91.00	-84.35
3.4	-12.18	-66.78	-65.14	-15.36	-70.54	-68.58	-19.93	-76.02	-73.76
3.6	-14.57	-55.06	-55.90	-17.09	-57.99	-58.66	-20.58	-62.13	-62.72
3.8	-14.96	-44.81	-47.15	-17.01	-47.11	-49.37	-19.71	-50.21	-52.51
4.0	-14.39	-36.23	-39.37	-16.08	-37.99	-41.17	-18.20	-40.33	-43.60

Table S5 Absolute energies (kJ·mol^{-1}) as function of Au^{I}-Ir^{I} distance (Å) of model systems **3** calculated at the RHF/def2-TZVPP, MP2/def2-TZVPP and PBE0-D3BJ/def2-TZVPP levels of theory with the counterpoise correction (cp) for the basis set superposition error (BSSE).

**R**	**3a**			**3b**			**3c**		
	ΔE_{int}^{RHF}	ΔE_{int}^{MP2}	ΔE_{int}^{PBE0}	ΔE_{int}^{RHF}	ΔE_{int}^{MP2}	ΔE_{int}^{PBE0}	ΔE_{int}^{RHF}	ΔE_{int}^{MP2}	ΔE_{int}^{PBE0}
2.4	426.61	207.94	224.22	448.65	222.88	240.04	466.46	230.54	250.41
2.6	183.95	16.90	28.62	200.33	27.41	39.58	215.96	34.60	48.28
2.8	64.24	-62.05	-55.10	76.01	-55.06	-47.95	88.44	-49.54	-41.67
3.0	6.73	-88.41	-85.44	15.08	-84.01	-80.96	24.57	-80.17	-76.82
3.2	-19.56	-91.55	-91.34	-13.62	-88.83	-88.58	-6.47	-86.36	-86.02
3.4	-30.35	-85.31	-86.78	-26.06	-83.72	-85.10	-20.64	-82.17	-83.60
3.6	-33.59	-76.11	-78.37	-30.38	-75.14	-77.36	-26.20	-74.22	-76.44
3.8	-33.25	-66.65	-69.12	-30.74	-65.98	-68.45	-27.43	-65.48	-67.91
4.0	-31.31	-57.95	-60.33	-29.25	-57.45	-59.83	-26.54	-57.11	-59.45

Table S6 Absolute energies (kJ·mol^{-1}) as function of Au^{I}-Ir^{I} distance (Å) of model systems **4** calculated at the RHF/def2-TZVPP, MP2/def2-TZVPP and PBE0-D3BJ/def2-TZVPP levels of theory with the counterpoise correction (cp) for the basis set superposition error (BSSE).

R	**4a**			**4b**			**4c**		
	ΔE_{int}^{RHF}	ΔE_{int}^{MP2}	ΔE_{int}^{PBE0}	ΔE_{int}^{RHF}	ΔE_{int}^{MP2}	ΔE_{int}^{PBE0}	ΔE_{int}^{RHF}	ΔE_{int}^{MP2}	ΔE_{int}^{PBE0}
2.4	184.36	-20.38	11.21	188.94	-20.92	11.63	191.53	-26.19	8.70
2.6	58.36	-104.68	-80.88	61.87	-106.02	-81.04	64.69	-110.33	-83.47
2.8	1.08	-127.78	-111.67	3.89	-129.33	-112.17	6.61	-133.13	-114.35
3.0	-22.95	-124.14	-114.56	-20.60	-125.65	-115.19	-18.02	-128.91	-117.07
3.2	-31.30	-110.54	-105.77	-29.25	-111.71	-106.32	-26.81	-114.60	-107.91
3.4	-32.53	-94.73	-93.01	-30.68	-95.77	-93.47	-28.36	-97.95	-94.73
3.6	-30.75	-79.75	-79.79	-29.04	-80.50	-80.08	-26.83	-82.13	-81.00
3.8	-27.90	-66.73	-67.70	-26.30	-67.20	-67.78	-24.18	-68.41	-68.41
4.0	-24.85	-55.86	-57.20	-23.34	-56.11	-57.15	-21.31	-56.94	-57.49

Table S7 Absolute energies (kJ·mol^{-1}) as function of Au^{I}-Ir^{I} distance (Å) of model systems **5** calculated at the RHF/def2-TZVPP, MP2/def2-TZVPP and PBE0-D3BJ/def2-TZVPP levels of theory with the counterpoise correction (cp) for the basis set superposition error (BSSE).

R	5a			5b			5c		
	ΔE_{int}^{RHF}	ΔE_{int}^{MP2}	ΔE_{int}^{PBE0}	ΔE_{int}^{RHF}	ΔE_{int}^{MP2}	ΔE_{int}^{PBE0}	ΔE_{int}^{RHF}	ΔE_{int}^{MP2}	ΔE_{int}^{PBE0}
2.4	323.64	110.88	146.36	318.50	104.10	140.71	302.02	87.24	125.85
2.6	169.52	4.18	30.63	166.99	-0.04	27.36	157.96	-9.96	18.83
2.8	89.54	-37.15	-19.33	88.45	-39.83	-21.25	83.78	-45.56	-26.02
3.0	47.46	-48.83	-37.87	47.16	-50.50	-38.99	45.01	-53.68	-41.55
3.2	24.98	-48.12	-42.01	25.09	-49.16	-42.59	24.36	-50.79	-43.81
3.4	12.77	-42.89	-39.92	13.10	-43.56	-40.17	13.17	-44.22	-40.58
3.6	6.08	-36.61	-35.52	6.50	-36.94	-35.56	6.99	-37.11	-35.48
3.8	2.39	-30.54	-30.63	2.86	-30.71	-30.54	3.54	-30.59	-30.17
4.0	0.39	-25.19	-25.94	0.86	-25.27	-25.82	1.62	-24.94	-25.27

Table S8 Absolute energies (kJ·mol^{-1}) as function of Au^{I}-Ir^{I} distance (Å) of model systems **6** calculated at the RHF/def2-TZVPP, MP2/def2-TZVPP and PBE0-D3BJ/def2-TZVPP levels of theory with the counterpoise correction (cp) for the basis set superposition error (BSSE).

R	6a			6b			6c		
	ΔE_{int}^{RHF}	ΔE_{int}^{MP2}	ΔE_{int}^{PBE0}	ΔE_{int}^{RHF}	ΔE_{int}^{MP2}	ΔE_{int}^{PBE0}	ΔE_{int}^{RHF}	ΔE_{int}^{MP2}	ΔE_{int}^{PBE0}
2.4	348.87	114.93	151.46	354.12	114.73	152.05	357.77	110.62	149.62
2.6	188.64	7.36	34.69	194.61	6.28	34.39	197.79	2.34	31.92
2.8	105.96	-35.94	-17.36	108.65	-37.53	-18.20	111.22	-41.25	-20.71
3.0	59.54	-49.33	-37.82	61.40	-51.17	-38.91	63.36	-54.64	-41.38
3.2	33.63	-49.79	-43.26	34.88	-51.67	-44.48	36.30	-54.85	-46.82
3.4	18.90	-45.27	-41.92	19.72	-47.03	-43.18	20.69	-49.96	-45.40
3.6	10.44	-39.20	-37.91	10.95	-40.84	-39.12	11.55	-43.47	-41.17
3.8	5.54	-33.10	-33.10	5.84	-34.56	-34.23	6.18	-36.94	-36.11
4.0	2.72	-27.57	-28.33	2.89	-28.87	-29.33	3.03	-31.00	-31.09

NBO Effective Charges

Table S9 Effective NBO charges for atoms coordinated to the metal centers, computed at the MP2/def2-TZVPP level of theory. Values in parentheses correspond to the isolated monomers.

Model	L(1)	L(2)	X	C	O	P(1)	P(2)
**1a**	-0.37(-0.43)	-0.37(-0.43)	-0.42 (-0.40)	+0.53 (+0.52)	-0.48 (-0.44)	+0.52 (+0.48)	+0.52 (+0.48)
**1b**	-0.36 (-0.43)	-0.36 (-0.43)	-0.36 (-0.33)	+0.53 (+0.52)	-0.48 (-0.43)	+0.53 (+0.48)	+0.53 (+0.48)
**1c**	-0.35 (-0.43)	-0.35 (-0.43)	-0.26 (-0.22)	+0.52 (+0.52)	-0.48 (-0.43)	+0.53 (+0.47)	+0.53 (+0.47)
**2a**	-1.10 (-1.10)	-1.04 (-1.10)	-0.42 (-0.40)	+0.55 (+0.52)	-0.47 (-0.44)	+0.51 (+0.48)	+0.51 (+0.48)
**2b**	-1.10 (-1.10)	-1.03 (-1.10)	-0.35 (-0.33)	+0.55 (+0.52)	-0.47 (-0.43)	+0.51 (+0.48)	+0.51 (+0.48)
**2c**	-1.10 (-1.10)	-1.02 (-1.10)	-0.25 (-0.22)	+0.54 (+0.52)	-0.47 (-0.43)	+0.51 (+0.47)	+0.51 (+0.47)
**3a**	-0.98 (-0.99)	-0.99 (-0.99)	-0.38 (-0.40)	+0.52 (+0.52)	-0.45 (-0.43)	+0.48 (+0.48)	+0.48 (+0.48)
**3b**	-0.98 (-0.99)	-0.99 (-0.99)	-0.39 (-0.33)	+0.52 (+0.52)	-0.45 (-0.43)	+0.48 (+0.48)	+0.48 (+0.48)
**3c**	-0.99 (-0.99)	-0.99 (-0.99)	-0.15 (-0.22)	+0.51 (+0.52)	-0.45 (-0.43)	+0.47 (+0.47)	+0.47 (+0.47)
**4a**	+0.23 (+0.23)	+0.25 (+0.23)	-0.34 (-0.40)	+0.53 (+0.52)	-0.41 (-0.44)	+0.23 (+0.48)	+0.25 (+0.48)
**4b**	+0.23 (+0.23)	+0.25 (+0.23)	-0.24 (-0.33)	+0.53 (+0.52)	-0.41 (-0.44)	+0.23 (+0.48)	+0.25 (+0.48)
**4c**	+0.24 (+0.23)	+0.25 (+0.23)	-0.09 (-0.22)	+0.53 (+0.52)	-0.40 (-0.44)	+0.23 (+0.47)	+0.24 (+0.47)
**5a**	-0.94 (-0.97)	-1.01 (-1.01)	-0.37 (-0.40)	+0.51 (+0.52)	-0.44 (-0.44)	+0.50 (+0.48)	+0.49 (+0.48)
**5b**	-0.94 (-0.97)	-1.01 (-1.01)	-0.30 (-0.33)	+0.51 (+0.52)	-0.43(-0.43)	+0.50 (+0.48)	+0.49 (+0.48)
**5c**	-0.93 (-0.97)	-1.01 (-1.01)	-0.17 (-0.22)	+0.51 (+0.52)	-0.43(-0.43)	+0.49 (+0.47)	+0.49 (+0.47)
**6a**	-1.03 (-1.06)	+0.26 (+0.23)	-0.36 (-0.40)	+0.51 (+0.52)	-0.44 (-0.44)	+0.49 (+0.48)	+0.49 (+0.48)
**6b**	-1.03 (-1.06)	+0.26 (+0.23)	-0.28 (-0.33)	+0.51 (+0.52)	-0.44 (-0.43)	+0.49 (+0.48)	+0.49 (+0.48)
**6c**	-1.04 (-1.06)	+0.26 (+0.23)	-0.14 (-0.22)	+0.51 (+0.52)	-0.43 (-0.43)	+0.49 (+0.47)	+0.49 (+0.47)

NEDA

Table S10 Natural energy decomposition analysis (NEDA) contributions to the total interaction energy at the MP2 potential energy curve minimum, computed at the PBE0-D3(BJ)/def2-TZVPP level of theory.[a]

Models	ΔE_{ES} (%)[a]	ΔE_{XC} (%)[a]	ΔE_{POL} (%)[a]	ΔE_{CT} (%)[a]	ΔE_{SE}	ΔE_{DEF}	ΔE_{EL}	ΔE_{CORE}	ΔE_{int}
**1a**	-181.1 (28.3)	-169.3 (26.5)	-143.8 (22.5)	-144.8 (22.7)	66.9	564.0	-257.9	327.8	-74.9
**1b**	-190.1 (28.1)	-177.6 (26.3)	-152.8 (22.6)	-155.3 (23.0)	71.0	595.3	-271.9	346.7	-80.5
**1c**	-203.5 (27.8)	-191.1 (26.1)	-160.0 (21.8)	-178.3 (24.3)	73.8	641.7	-289.7	376.8	-91.1
**2a**	-169.0 (22.4)	-204.5 (27.1)	-183.9 (24.4)	-196.7 (26.1)	87.8	679.0	-265.0	386.7	-75.0
**2b**	-176.7 (22.3)	-213.5 (26.9)	-196.2 (24.8)	-206.3 (26.0)	93.7	712.3	-279.2	405.1	-80.5
**2c**	-187.3 (22.2)	-227.4 (27.0)	-203.0 (24.1)	-224.3 (26.6)	96.6	752.9	-293.7	428.9	-89.1
**3a**	-109.7 (24.1)	-117.0 (25.9)	-98.6 (21.9)	-125.5 (27.8)	50.6	359.5	-157.7	191.9	-91.3
**3b**	-102.1 (23.1)	-115.7 (26.2)	-100.7 (22.8)	-123.3 (27.9)	51.6	353.3	-151.2	186.0	-88.5
**3c**	-95.1 (22.4)	-115.7 (27.2)	-90.7 (21.3)	-124.1 (29.2)	46.5	339.5	-139.3	177.4	-86.1
**4a**	-147.3 (20.8)	-190.2 (26.9)	-189.0 (26.7)	-181.3 (25.6)	95.2	593.7	-241.1	308.3	-114.2
**4b**	-145.9 (20.3)	-193.7(26.9)	-195.6 (27.2)	-184.3 (25.6)	98.5	604.8	-243.1	312.7	-114.7
**4c**	-146.2 (20.1)	-198.9 (27.4)	-187.7 (25.9)	-193.0 (26.6)	94.4	609.2	-239.5	315.8	-116.6
**5a**	-94.9 (20.7)	-141.6 (31.0)	-112.6 (24.6)	-108.3 (23.7)	55.6	416.7	-151.9	219.6	-40.6
**5b**	-95.7 (20.4)	-145.5 (31.0)	-117.6 (25.1)	-110.1 (23.5)	58.1	427.6	-155.2	224.0	-41.3
**5c**	-101.0 (20.5)	-155.2 (31.6)	-117.9 (24.0)	-117.6 (23.9)	58.2	449.3	-160.7	235.9	-42.4
**6a**	-91.5 (18.4)	-156.1 (31.4)	-132.9 (26.7)	-117.3 (23.6)	65.8	455.6	-158.5	233.7	-42.1
**6b**	-94.7 (18.2)	-162.3 (31.2)	-142.1 (27.3)	-120.9 (23.2)	70.5	476.7	-166.3	243.9	-43.3
**6c**	-101.2 (18.5)	-172.8 (31.6)	-144.1 (26.3)	-129.0 (23.6)	71.5	501.5	-173.8	257.2	-45.6

[a] The percentage contributions are computed with respect to the total stabilizing energy, defined as the sum of ΔE_{ES}, ΔE_{XC}, ΔE_{POL}, and ΔE_{CT}, according to **Eq S7**.

IGMH Analysis

Table S11 IGMH results for metal atoms in model systems **1** at the MP2/def2-TZVPP level of theory. Values of the IGMH atomic *δg* index (δg^{atom}) and the intrinsic bond strength index for weak interactions (IBSIW) are reported in atomic units (a.u.).

Au^{I}···Ir^{I}						
	**1a**		**1b**		**1c**	
	**Au**	**Ir**	**Au**	**Ir**	**Au**	**Ir**
δg^{atom} (a.u.)	0.71	0.39	0.72	0.41	0.75	0.44
δg^{atom} (%)	64	36	64	36	65	37

Table S12 IGMH results for metal atoms in model systems **2** at the MP2/def2-TZVPP level of theory. Values of the IGMH atomic *δg* index (δg^{atom}) and the intrinsic bond strength index for weak interactions (IBSIW) are reported in atomic units (a.u.).

Au^{I}···Ir^{I}						
	**2a**		**2b**		**2c**	
	**Au**	**Ir**	**Au**	**Ir**	**Au**	**Ir**
δg^{atom} (a.u.)	0.68	0.58	0.69	0.60	0.72	0.63
δg^{atom} (%)	43	36	42	36	42	37

Table S13 IGMH results for metal atoms in model systems **3** at the MP2/def2-TZVPP level of theory. Values of the IGMH atomic *δg* index (δg^{atom}) and the intrinsic bond strength index for weak interactions (IBSIW) are reported in atomic units (a.u.).

Au^{I}···Ir^{I}						
	**3a**		**3b**		**3c**	
	**Au**	**Ir**	**Au**	**Ir**	**Au**	**Ir**
δg^{atom} (a.u.)	0.42	0.35	0.41	0.34	0.41	0.33
δg^{atom} (%)	40	33	40	33	40	32

Table S14 IGMH results for metal atoms in model systems **4** at the MP2/def2-TZVPP level of theory. Values of the IGMH atomic *δg* index (δg^{atom}) and the intrinsic bond strength index for weak interactions (IBSIW) are reported in atomic units (a.u.).

	Au^{I}···Ir^{I}					
	**4a**		**4b**		**4c**	
	**Au**	**Ir**	**Au**	**Ir**	**Au**	**Ir**
δg^{atom} (a.u.)	0.67	0.47	0.68	0.48	0.70	0.48
δg^{atom} (%)	52	37	53	37	53	36

Table S15 IGMH results for metal atoms in model systems **5** at the MP2/def2-TZVPP level of theory. Values of the IGMH atomic *δg* index (δg^{atom}) and the intrinsic bond strength index for weak interactions (IBSIW) are reported in atomic units (a.u.).

	Au^{I}···Ir^{I}					
	**5a**		**5b**		**5c**	
	**Au**	**Ir**	**Au**	**Ir**	**Au**	**Ir**
δg^{atom} (a.u.)	0.51	0.40	0.52	0.40	0.54	0.44
δg^{atom} (%)	43	33	43	33	43	35

Table S16 IGMH results for metal atoms in model systems **6** at the MP2/def2-TZVPP level of theory. Values of the IGMH atomic *δg* index (δg^{atom}) and the intrinsic bond strength index for weak interactions (IBSIW) are reported in atomic units (a.u.).

	Au^{I}···Ir^{I}					
	**6a**		**6b**		**6c**	
	**Au**	**Ir**	**Au**	**Ir**	**Au**	**Ir**
δg^{atom} (a.u.)	0.50	0.42	0.51	0.43	0.54	0.45
δg^{atom} (%)	40	33	40	33	39	33

Table S17 IGMH analysis results for the additional BCPs in model systems **3**, calculated at the MP2/def2-TZVPP level of theory. Values of the IGMH atomic pair index (δg^{pair}) and the intrinsic bond strength index for weak interactions (IBSIW) are reported in atomic units (a.u.).

	**3a**		**3b**		**3c**	
BCP	**N-H···Cl-Ir**	**N-H···O-C**	**N-H···Br-Ir**	**N-H···O-C**	**N-H···I-Ir**	**N-H···O-C**
δg^{pair} (a.u.)	0.10	0.04	0.09	0.05	0.10	0.05
δg^{pair} (%)	9	4	9	4	10	5
IBSIW (a.u.)	1.88	0.90	1.53	0.93	1.40	0.98

Table S18 IGMH analysis results for the additional BCPs in model systems **4**, calculated at the MP2/def2-TZVPP level of theory. Values of the IGMH atomic pair index (δg^{pair}) and the intrinsic bond strength index for weak interactions (IBSIW) are reported in atomic units (a.u.).

	**P-H···X-Ir**		
	**4a**	**4b**	**4c**
δg^{pair} (a.u.)	0.08	0.08	0.08
δg^{pair} (%)	6	6	6
IBSIW (a.u.)	0.73	0.64	0.55

Table S19 IGMH analysis results for the additional BCPs in model systems **5**, calculated at the MP2/def2-TZVPP level of theory. Values of the IGMH atomic pair index (δg^{pair}) and the intrinsic bond strength index for weak interactions (IBSIW) are reported in atomic units (a.u.).

	**5a**		**5b**		**5c**	
BCP	**P-H···C-Au**	**C-H···Cl-Ir**	**P-H···C-Au**	**C-H···Br-Ir**	**P-H···C-Au**	**C-H···I-Ir**
δg^{pair} (a.u.)	0.10	0.05	0.10	0.04	0.09	0.05
δg^{pair} (%)	8	4	8	4	7	4
IBSIW (a.u.)	0.86	0.56	0.81	0.48	0.73	0.51

Table S20 IGMH analysis results for the additional BCPs in model systems **6**, calculated at the MP2/def2-TZVPP level of theory. Values of the IGMH atomic pair index (δg^{pair}) and the intrinsic bond strength index for weak interactions (IBSIW) are reported in atomic units (a.u.).

	**6a**		**6b**	**6c**
BCP	**C-H···Cl-Ir**	**P-H···C-Au**	**P-H···C-Au**	**P-H···C-Au**
δg^{pair} (a.u.)	0.03	0.03	0.03	0.03
δg^{pair} (%)	2.48	1.98	1.95	2.09
IBSIW (a.u.)	0.33	0.31	0.32	0.37

Cartesian Coordinates

Model systems 1a

MP2 optimization

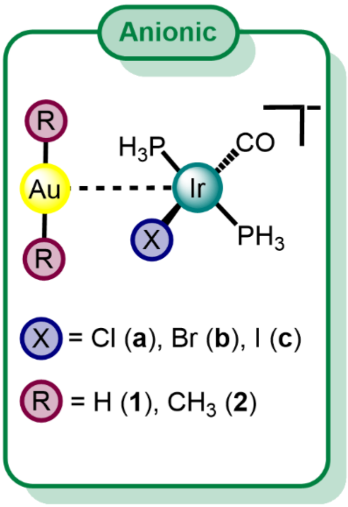

Ir	-2.59094100	-1.05599000	-1.36439000
Au	-2.61107000	-1.44276400	1.42537500
P	-0.35660900	-1.24807700	-1.26115700
H	0.10691000	-2.45144400	-0.72838200
H	0.42106900	-1.19921300	-2.43607600
H	0.35428900	-0.32121100	-0.49483200
C	-2.51721100	0.73114400	-1.35451500
O	-2.46861500	1.90822900	-1.37034200
Cl	-2.68633000	-3.38123500	-1.73912500
P	-4.83380100	-1.06332800	-1.26787000
H	-5.60128900	-0.95070700	-2.44510600
H	-5.39648200	-2.22434800	-0.73663100
H	-5.46821100	-0.08094600	-0.50356600
H	-0.99319200	-1.51549000	1.45536300
H	-4.22952000	-1.38191600	1.45048100

RHF optimization

Ir	-1.79566400	-1.21626100	-1.74128800
Au	-5.88749800	-0.60658000	3.21998800
P	-0.17417100	-1.21301800	-3.42311600
H	0.56167800	-2.38705100	-3.57881700
H	-0.63423300	-1.05043100	-4.72924600
H	0.87428400	-0.28700600	-3.43713900
C	-1.06693500	0.22023700	-0.82984300
O	-0.60830300	1.09199600	-0.29375600
P	-3.56829300	-1.46614800	-0.18891300
H	-4.84284600	-1.36085600	-0.73082800
H	-3.63728500	-2.70824400	0.42889000
H	-3.66286200	-0.61205400	0.90170000
H	-5.72042800	0.56564700	4.40714800
H	-6.04506900	-1.78276300	2.01836200
Cl	-2.71510200	-3.09671200	-2.97028200

RI-SCS-MP2-ZORA optimization

Ir	-2.59207979208438	-1.05722439356387	-1.43104256331421
Au	-2.60528016645494	-1.38920043897012	1.48734691074331
P	-0.33757381599959	-1.26997712324646	-1.29836090692366
H	0.11789964620756	-2.44453684505561	-0.68415782667178
H	0.44317750104634	-1.31275708004373	-2.48045160305910
H	0.39659932858067	-0.30246189190313	-0.59275383788070
C	-2.52018112100166	0.73021800493725	-1.27020910640753
O	-2.47392673308151	1.90158750081636	-1.19023323455450
Cl	-2.68080804405543	-3.37800657638412	-1.88284828612349
P	-4.85533860235938	-1.07536081153858	-1.30723950353193
H	-5.63540838194073	-1.01308574263413	-2.48902550101555
H	-5.41799396178785	-2.21944515239189	-0.72512196512197
H	-5.49937733312145	-0.06419293973410	-0.57512749870676
H	-0.97305142173023	-1.42023715409784	1.54224320994981
H	-4.23766010221737	-1.36261535619002	1.52620871261806

SCS-MP2-ZORA^{NR} optimization

Ir	-2.59156868550456	-1.07706516633213	-1.48287753024495
Au	-2.60249985299298	-1.24130284903527	1.59244488941111
P	-0.26723215742396	-1.30518317347629	-1.35071412592008
H	0.16542178799247	-2.47461922050555	-0.71264920858600
H	0.49544922430045	-1.40016585837568	-2.54169842618857
H	0.50830840742416	-0.34861237241681	-0.67539581757409
C	-2.51669515783511	0.73376031494350	-1.10810373727577
O	-2.47021150709318	1.87800930147369	-0.88870481502556
Cl	-2.68820694477942	-3.40011657348890	-2.11690527578998
P	-4.92733200670785	-1.11018623885826	-1.35482275492933
H	-5.69123267176424	-1.14163928778155	-2.54851529506205
H	-5.45859608086539	-2.23928139011735	-0.71863044552421
H	-5.62234327118841	-0.09176347921253	-0.68236578042617
H	-0.83297583781568	-1.30712015111285	1.61350856575348
H	-4.37128824574626	-1.15200985570400	1.60465675738220

Model system 1b

MP2 optimization

Ir	-2.59094100	-1.05599000	-1.36439000
Au	-2.61107000	-1.44276400	1.42537500
P	-0.35660900	-1.24807700	-1.26115700
H	0.10691000	-2.45144400	-0.72838200
H	0.42106900	-1.19921300	-2.43607600
H	0.35428900	-0.32121100	-0.49483200
C	-2.51721100	0.73114400	-1.35451500
O	-2.46861500	1.90822900	-1.37034200
Cl	-2.68633000	-3.38123500	-1.73912500
P	-4.83380100	-1.06332800	-1.26787000
H	-5.60128900	-0.95070700	-2.44510600
H	-5.39648200	-2.22434800	-0.73663100
H	-5.46821100	-0.08094600	-0.50356600
H	-0.99319200	-1.51549000	1.45536300
H	-4.22952000	-1.38191600	1.45048100

RHF optimization

Ir	-1.81025200	-1.23324800	-1.74588100
Au	-5.84475100	-0.56842100	3.22000000
P	-0.16451100	-1.18852300	-3.40460400
H	0.59659300	-2.34526100	-3.56541800
H	-0.59990900	-1.00808000	-4.71649400
H	0.86660300	-0.24307600	-3.37810500
C	-1.09764100	0.20240200	-0.81886000
O	-0.64869800	1.07288700	-0.27430000
P	-3.58594000	-1.48857300	-0.19673300
H	-4.86140100	-1.38069200	-0.73562400
H	-3.65522900	-2.72865000	0.42476000
H	-3.67448600	-0.63092400	0.89159400
H	-5.64161700	0.61801100	4.38694100
H	-6.03787100	-1.75905800	2.03776400
Br	-2.76361700	-3.22803900	-3.07218000

Model system 1c

MP2 optimization

Ir	-2.59042900	-1.04097700	-1.28423300
Au	-2.61529400	-1.54359000	1.43107600
P	-0.35586300	-1.22562100	-1.21499300
H	0.16167900	-2.40114200	-0.66984000
H	0.38288100	-1.18350600	-2.41399300
H	0.36286700	-0.26722700	-0.49149600
C	-2.51648200	0.74564600	-1.43351900
O	-2.46799000	1.91609800	-1.56413400
P	-4.83280700	-1.04088200	-1.22168600
H	-5.56198100	-0.93818400	-2.42288300
H	-5.44706300	-2.16970400	-0.67823400
H	-5.47228700	-0.02644700	-0.50021300
H	-0.99869600	-1.61265200	1.45632400
H	-4.23215600	-1.47924300	1.45155900
I	-2.69680600	-3.63953200	-1.85152300

RHF optimization

Ir	-1.82974500	-1.25782800	-1.75398100
Au	-5.79083200	-0.52092600	3.21789900
P	-0.15269300	-1.15761100	-3.37946200
H	0.64254400	-2.28984900	-3.54733700
H	-0.55311000	-0.95103300	-4.69837700
H	0.85349400	-0.18832600	-3.29723800
C	-1.13553500	0.17680000	-0.80644300
O	-0.69789800	1.04500400	-0.25121000
P	-3.60787100	-1.51503100	-0.20610300
H	-4.88594100	-1.40097200	-0.73713500
H	-3.68048200	-2.75074600	0.42334500
H	-3.68383500	-0.65078600	0.87802500
H	-5.54291900	0.68151000	4.35912400
H	-6.02816000	-1.72794400	2.06034800
I	-2.82974500	-3.40150500	-3.20859500

Model system 2a

MP2 optimization

Ir	-2.27130400	-1.15669000	-1.19271700
Au	-3.00987300	-1.57452000	1.50043900
P	-0.13431300	-1.46284400	-1.70754200
H	0.70579400	-2.21883600	-0.86241100
H	0.12840100	-2.14452200	-2.90423400
H	0.71573200	-0.36088000	-1.88769600
C	-2.02224400	0.58342100	-0.86029500
O	-1.85207900	1.73105500	-0.66835300
Cl	-2.62505600	-3.37905100	-1.93099300
P	-4.51805100	-0.96078700	-1.25047000
H	-5.12282500	-0.86515000	-2.51936100
H	-5.25418600	-2.03336800	-0.74219200
H	-5.15250900	0.11653000	-0.62696600
C	-4.72448400	-0.50235100	1.96598500
H	-4.67772800	0.53636200	1.62176700
H	-5.65301800	-0.93334600	1.57677600
H	-4.83426800	-0.46668300	3.05524800
C	-1.27516000	-2.67133300	1.36560700
H	-1.22076800	-3.24226900	2.30244900
H	-1.23959600	-3.38428700	0.54065000
H	-0.38186700	-2.04220000	1.31885300

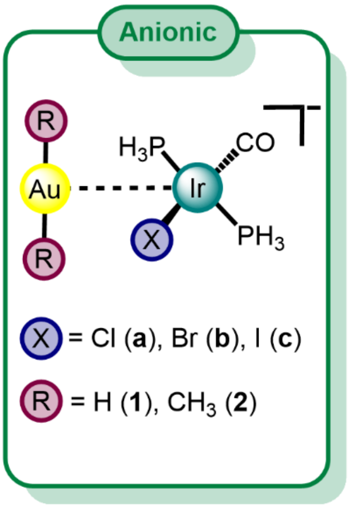

RHF optimization

Ir	0.22443000	-0.04461400	-0.23104700
Au	1.97294900	0.19881300	5.85710100
P	-0.14446600	-0.13001800	-2.53397900
H	-0.65921400	-1.32447200	-3.03605400
H	-1.08406800	0.76414000	-3.04523700
H	0.88808100	0.07600600	-3.45473200
C	2.03352600	0.32400700	-0.38745200
O	3.12575900	0.54477100	-0.50796800
P	0.28719000	-0.02220700	2.13775100
H	-0.57392100	0.89230900	2.73363500
H	-0.12432100	-1.20797700	2.73726400
H	1.46999900	0.23424100	2.81890000
C	2.14153200	2.15329700	4.99350400
H	2.81063400	2.18162200	4.12981300
H	1.18130500	2.55369900	4.65888000
H	2.53906100	2.87296100	5.71071400
C	1.80618100	-1.74768100	6.71856200
H	0.77515600	-2.10568900	6.74287900
H	2.38403600	-2.49597600	6.17259300
H	2.16672200	-1.76677700	7.74869500
Cl	-2.14908200	-0.52863800	-0.07570800

RI-SCS-MP2-ZORA optimization

Ir	-2.37964332088868	-1.10723995172065	-1.35682629222228
Au	-2.87076637255335	-1.60135689381865	1.53896405541688
P	-0.14948656797247	-1.26678044954069	-1.59755248872726
H	0.54109738182025	-2.16924633629298	-0.76370589935415
H	0.35364347608353	-1.69635285591634	-2.84314552298109
H	0.67017917909473	-0.13265077859616	-1.41897231501983
C	-2.29795890919291	0.63610534189784	-0.93014399010388
O	-2.23521367499117	1.78108928284327	-0.68165388242313
Cl	-2.49848173741055	-3.34186487417185	-2.14656197862036
P	-4.65573795904211	-1.14280103975495	-1.32580943535669
H	-5.32553933539241	-1.22550527089182	-2.56903241924660
H	-5.25994910116010	-2.23098132602681	-0.67671502708347
H	-5.37316057158960	-0.07558486208298	-0.76115966164345
C	-4.40213117180700	-0.22701879053035	1.91578692715487
H	-4.29687843128127	0.71840335827277	1.35603133447270
H	-5.41847459707963	-0.61331860338434	1.71662610769850
H	-4.38633185313769	0.04460745726315	2.98591135079607
C	-1.33376020303794	-2.98889537073346	1.41477361127222
H	-1.40590911434296	-3.69569759175643	2.26074398948031
H	-1.35657285814249	-3.58282386903854	0.48929354357182
H	-0.33832625797612	-2.51383557601997	1.48769199291882

SCS-MP2-ZORA^{NR} optimization

Ir	-2.43610100000000	-1.23325200000000	-1.47720800000000
Au	-2.81551400000000	-1.62715200000000	1.55196800000000
P	-0.21312200000000	-1.24406100000000	-1.51763800000000
H	0.49458600000000	-2.22284600000000	-0.80191300000000
H	0.47400500000000	-1.28572700000000	-2.74369000000000
H	0.40638700000000	-0.09866200000000	-0.97870300000000
C	-2.38250000000000	0.49903300000000	-1.04348300000000
O	-2.39660700000000	1.65625800000000	-0.84704400000000
Cl	-2.55911300000000	-3.47527900000000	-2.16601200000000
P	-4.74295100000000	-1.14390800000000	-1.39930300000000
H	-5.49336300000000	-1.03841400000000	-2.59725500000000
H	-5.46938800000000	-2.17095200000000	-0.77541300000000
H	-5.31547500000000	-0.03442200000000	-0.75233000000000
C	-4.24013600000000	-0.20188300000000	1.84704600000000
H	-4.11206900000000	0.70164300000000	1.26141000000000
H	-5.27667400000000	-0.59065200000000	1.70175600000000
H	-4.18587600000000	0.08506500000000	2.90766200000000
C	-1.26360300000000	-3.02835200000000	1.46284600000000
H	-1.33213900000000	-3.67110400000000	2.35205900000000
H	-1.28359900000000	-3.73369000000000	0.60436500000000
H	-0.27615000000000	-2.57339200000000	1.50542400000000

Model system 2b

MP2 optimization

Ir	-2.27501300	-1.15605200	-1.17856400
Au	-3.00620300	-1.56408600	1.50075400
P	-0.14596200	-1.49764700	-1.70688100
H	0.63038700	-2.39910300	-0.95006900
H	0.12299700	-2.02633200	-2.97759100
H	0.75884100	-0.42378800	-1.72600200
C	-2.00773600	0.59040200	-0.88863700
O	-1.82349000	1.74003600	-0.72464600
P	-4.51650200	-0.93137500	-1.24971400
H	-5.11280300	-0.83647700	-2.52239000
H	-5.28111500	-1.98001800	-0.73292300
H	-5.13383500	0.16523200	-0.64084600
C	-4.75051100	-0.54422500	1.97368100
H	-4.72821100	0.50141200	1.64900200
H	-5.66518700	-0.99146900	1.57093700
H	-4.86711600	-0.53142100	3.06274100
C	-1.24152900	-2.61276300	1.37581100
H	-1.16296300	-3.13128700	2.34153000
H	-1.20079800	-3.36732600	0.58939000
H	-0.36337800	-1.96758900	1.28545700
Br	-2.65400300	-3.49757400	-1.96636100

RHF optimization

Ir	-1.57984200	-1.39169700	-2.60599800
Au	-3.95765000	-1.16169200	3.23497600
P	-0.51417300	-1.04525100	-4.65363500
H	0.48426800	-1.95304700	-5.00288800
H	-1.31080200	-1.11810500	-5.79512500
H	0.16135000	0.14928000	-4.92532700
C	-1.10330200	0.27093400	-1.93600100
O	-0.79995000	1.27933200	-1.55570300
P	-2.73389300	-1.98903900	-0.62361600
H	-4.07717000	-2.30119300	-0.80128100
H	-2.26850700	-3.14845700	-0.01156900
H	-2.79722700	-1.11394100	0.45359700
C	-4.97562700	0.18399300	1.91261600
H	-4.30117400	0.88405200	1.41305700
H	-5.53012600	-0.33295200	1.12539600
H	-5.70424100	0.79348300	2.44910100
C	-2.94553600	-2.50058300	4.55428900
H	-3.09811600	-3.54707200	4.28356600
H	-1.86649400	-2.33539200	4.56805500
H	-3.29039400	-2.39187500	5.58421500
Br	-2.21552300	-3.69222900	-3.58304500

Model system 2c

MP2 optimization

Ir	-2.27910200	-1.16136600	-1.15684600
Au	-3.00524000	-1.57034200	1.49659400
P	-0.15946900	-1.54532500	-1.69869500
H	0.58711200	-2.51043800	-0.99294500
H	0.11645200	-1.99802100	-2.99740000
H	0.77323100	-0.49507500	-1.64450300
C	-1.97897500	0.59556500	-0.94516500
O	-1.77375700	1.74816600	-0.83592800
P	-4.51064400	-0.88301300	-1.25019300
H	-5.09506500	-0.78269200	-2.52751100
H	-5.32510700	-1.88776100	-0.72209800
H	-5.09156300	0.24635000	-0.66239800
C	-4.78625600	-0.62442200	1.98525400
H	-4.80474900	0.42685800	1.68007900
H	-5.68233200	-1.10001200	1.57424000
H	-4.90206500	-0.63563200	3.07443000
C	-1.20356100	-2.55461100	1.38209000
H	-1.11065900	-3.02130500	2.37345200
H	-1.14255700	-3.34681500	0.63540300
H	-0.34474700	-1.88971000	1.25945000
I	-2.73345500	-3.65013600	-2.00185900

RHF optimization

Ir	-1.64038600	-1.45965800	-2.59677900
Au	-3.88203400	-1.08738500	3.20833500
P	-0.52645100	-0.90717100	-4.57217800
H	0.48459700	-1.76722900	-4.99704300
H	-1.28906100	-0.84224200	-5.73690200
H	0.15402000	0.31057900	-4.68085800
C	-1.15476000	0.13333400	-1.77004200
O	-0.84306000	1.09875000	-1.30022700
P	-2.82845400	-2.19483300	-0.68080400
H	-4.17951100	-2.45159800	-0.88761100
H	-2.40216700	-3.41488700	-0.16482700
H	-2.87139300	-1.40808800	0.46462000
C	-4.87846900	0.17614600	1.79137300
H	-4.18892100	0.79104000	1.20740900
H	-5.48350000	-0.38464200	1.07448100
H	-5.55864700	0.86971000	2.28765400
C	-2.89252900	-2.34351900	4.62229900
H	-3.08715000	-3.40308500	4.44603900
H	-1.80835700	-2.21743800	4.60394600
H	-3.21337500	-2.13359000	5.64427300
I	-2.33452000	-3.82564500	-3.87847600

Model system 3a

MP2 optimization

Ir	-2.49134000	-1.15700300	-1.37241600
Au	-2.69292300	-1.54112900	1.67481000
P	-0.27667000	-0.66605000	-1.35593200
H	0.56646900	-1.51109200	-0.62569000
H	0.33040600	-0.72799000	-2.61381900
H	0.15577100	0.58645800	-0.91271400
C	-2.95848300	0.52322800	-0.97694800
O	-3.26731300	1.60515000	-0.64061600
N	-3.22599100	0.34668200	2.20347400
H	-3.30647700	0.97089700	1.40303100
H	-4.11982600	0.35755700	2.68630900
N	-2.17019600	-3.46668800	1.34921500
H	-1.31023300	-3.71325100	1.83085400
H	-2.88191800	-4.11378900	1.67635100
H	-2.54967600	0.75774900	2.84050700
H	-2.02930500	-3.63259400	0.34610800
Cl	-1.88472700	-3.35740800	-1.83790000
P	-4.63433000	-1.77676900	-1.78418000
H	-4.89766400	-2.06047900	-3.12761700
H	-5.09303400	-2.95368700	-1.18194500
H	-5.68194200	-0.90154000	-1.48633800

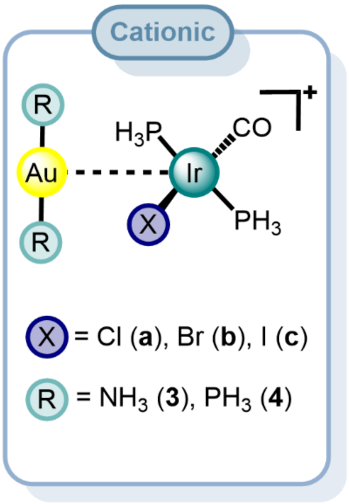

RHF optimization

Ir	-2.52639300	-0.47107600	-2.68476900
Au	-2.66968800	-2.39230500	3.49021600
P	-0.26223700	0.08090600	-2.34215000
H	-0.01415600	1.04546200	-1.36831000
H	0.56519500	-0.94709100	-1.89481600
H	0.50671700	0.57916700	-3.39238500
C	-2.46691300	0.01767400	-4.48378600
O	-2.43091800	0.30802600	-5.55908800
N	-3.14277400	-1.13337800	5.12158000
H	-3.35110500	-0.20450900	4.80871900
H	-3.94135000	-1.46353900	5.62827600
N	-2.20530100	-3.61079500	1.84587500
H	-1.28207100	-3.99391400	1.89934200
H	-2.84078300	-4.37737600	1.74335700
H	-2.38440300	-1.06569300	5.77255400
H	-2.26003600	-3.04867400	1.01056000
Cl	-2.60590700	-1.11165900	-0.31136800
P	-4.80930300	-1.04044000	-2.79713100
H	-5.13738600	-2.35340500	-2.46544900
H	-5.65082700	-0.34461100	-1.93229700
H	-5.50976400	-0.90451800	-3.99438900

RI-SCS-MP2-ZORA optimization

Ir	-2.50370702907624	-1.17818322723980	-1.40720375117652
Au	-2.67640422913531	-1.53482109906860	1.72013146183556
P	-0.25881806550105	-0.70668000656074	-1.37619312336333
H	0.59665603565231	-1.58427554914191	-0.68705227708351
H	0.34664646524398	-0.70747435208639	-2.64382981866843
H	0.18408026017929	0.53009584327259	-0.87826168559078
C	-2.94600294096404	0.51774753070348	-1.01589958953864
O	-3.24305630004459	1.60226808493031	-0.68285978194991
N	-3.27215227893841	0.35325586726747	2.24957458753934
H	-3.34836185868144	0.98689581601764	1.45080006725778
H	-4.18280210799197	0.33348348535493	2.71153300122643
N	-2.08913892503284	-3.46177048369707	1.41049842161210
H	-1.19530117624715	-3.66204650796359	1.86175145455346
H	-2.76604957061757	-4.12790169697971	1.78593700200267
H	-2.61700476550552	0.77256303870892	2.91150448720094
H	-1.98710581198656	-3.64515641211650	0.40273530581265
Cl	-1.94855675177871	-3.41406250999640	-1.85263856762148
P	-4.67899650928519	-1.74704538641512	-1.84687085032303
H	-4.95088473434022	-1.94987812135523	-3.21005045981673
H	-5.16893850999230	-2.94774528265291	-1.30547559081900
H	-5.71350319595642	-0.86101703098134	-1.50358629308956

SCS-MP2-ZORA^{NR} optimization

Ir	-2.48899945144935	-1.10219203858375	-1.53783642889109
Au	-2.68372384075162	-1.46490087088060	1.38672323963104
P	-0.18131747462108	-0.63389476724613	-1.44157921086391
H	0.61897663411661	-1.50459032185536	-0.68387012272051
H	0.45621611664342	-0.72441721511204	-2.69024081832984
H	0.30130521929405	0.60984935331567	-0.99971084710771
C	-2.97675757158804	0.65428611608071	-1.14159097695657
O	-3.28203511466139	1.73184090914989	-0.83763490058889
N	-3.30027273709025	0.55809342369379	2.43762208551491
H	-3.42519969577747	1.30695548722388	1.75835818775768
H	-4.17529828612578	0.47884353360411	2.95324883407427
N	-2.11389301743214	-3.73361857766746	1.44206106880108
H	-1.26514742692525	-3.94864124207055	1.96243821369750
H	-2.84016131131902	-4.34806613853326	1.80590208816569
H	-2.60159016870477	0.87958402861160	3.10568300630126
H	-1.95166283464483	-3.99058742766436	0.46583614495250
Cl	-1.87151949621253	-3.36797284118582	-1.92776915735194
P	-4.71507461173071	-1.79242740783754	-1.88754181780809
H	-4.99223615989782	-2.11641017929708	-3.22620838448966
H	-5.12841018635082	-2.97342948752919	-1.24948984199004
H	-5.80260058477117	-0.95005233621648	-1.59985636179770

Model system 3b

MP2 optimization

Ir	-2.48976200	-1.15810800	-1.38569800
Au	-2.69546700	-1.53359200	1.68123600
P	-0.27606300	-0.66740000	-1.36590100
H	0.56742700	-1.49188500	-0.61280600
H	0.33968400	-0.75174400	-2.61808500
H	0.14888400	0.59578500	-0.94466400
C	-2.95856300	0.51473700	-0.95379400
O	-3.26754500	1.59293600	-0.60595700
N	-3.22885900	0.35213800	2.21748300
H	-3.31058200	0.98036800	1.42030000
H	-4.12206600	0.35972900	2.70150700
N	-2.17312300	-3.46097200	1.36542800
H	-1.31372400	-3.70480900	1.84987200
H	-2.88603700	-4.10561900	1.69541900
H	-2.55192800	0.75993700	2.85593400
H	-2.02995700	-3.63713200	0.36517700
P	-4.63243700	-1.77772500	-1.79394600
H	-4.89369600	-2.08553800	-3.13231000
H	-5.10539700	-2.93777200	-1.17024400
H	-5.67460400	-0.88848700	-1.51681700
Br	-1.84722100	-3.45321700	-1.97168200

RHF optimization

Ir	-2.55105000	-0.37775700	-2.72549100
Au	-2.63460100	-2.52505200	3.54901200
P	-0.28892000	0.19202800	-2.39568800
H	-0.04041200	1.16752300	-1.43337100
H	0.55013500	-0.82599400	-1.94829300
H	0.46584100	0.68727300	-3.45791400
C	-2.49037100	0.09486800	-4.52953000
O	-2.45367600	0.37535900	-5.60700900
N	-3.12326400	-1.22392000	5.14183600
H	-3.32215400	-0.30308200	4.80016700
H	-3.93075400	-1.53623500	5.64565900
N	-2.15500500	-3.78168700	1.93418200
H	-1.22997900	-4.15874700	1.99996200
H	-2.78687800	-4.55294400	1.84433100
H	-2.37380100	-1.14216000	5.80141200
H	-2.20800600	-3.23829100	1.08801200
P	-4.83107700	-0.95800600	-2.84974500
H	-5.15769200	-2.27117500	-2.51890500
H	-5.68431300	-0.26148500	-1.99754300
H	-5.51746900	-0.82762000	-4.05602900
Br	-2.63758900	-1.03126200	-0.20460400

Model system 3c

MP2 optimization

Ir	-2.48181700	-1.18356200	-1.40221700
Au	-2.70000000	-1.51702600	1.68689500
P	-0.27140100	-0.68426500	-1.37465100
H	0.58303300	-1.50290000	-0.62739700
H	0.35497500	-0.73990100	-2.62309400
H	0.13497300	0.57940600	-0.93401000
C	-2.95294300	0.48461100	-0.93634100
O	-3.26208000	1.56140900	-0.58440700
N	-3.23379100	0.36817300	2.22546000
H	-3.31610500	0.99746400	1.42915400
H	-4.12662200	0.37408200	2.71011200
N	-2.17828700	-3.44801200	1.39033900
H	-1.31971700	-3.68735300	1.87896800
H	-2.89292900	-4.08875000	1.72489100
H	-2.55649100	0.77460300	2.86432500
H	-2.03213800	-3.63829200	0.39395700
P	-4.62709400	-1.79505200	-1.80205800
H	-4.91204400	-2.08308400	-3.13992700
H	-5.11073100	-2.95492800	-1.18611000
H	-5.65721400	-0.89771900	-1.50236700
I	-1.79313600	-3.59674200	-2.14903800

RHF optimization

Ir	-2.61038200	0.21831100	-2.54143600
Au	-2.56526700	-3.31591000	3.32895400
P	-0.33330700	0.70961600	-2.18441200
H	-0.00007600	1.28311700	-0.95984100
H	0.54203100	-0.37293500	-2.20720800
H	0.32227700	1.58120000	-3.05319600
C	-2.68088300	1.33521200	-4.03724300
O	-2.72336400	2.00211300	-4.92793300
N	-3.06411100	-1.27359100	3.44521500
H	-3.98574100	-1.12039500	3.80516400
H	-2.42827800	-0.75217800	4.01643500
N	-2.05741700	-5.36002500	3.15202200
H	-1.24145200	-5.59189400	3.68473300
H	-2.79722000	-5.95923800	3.46343800
H	-3.02876600	-0.88981100	2.51643900
H	-1.86653700	-5.59254600	2.19599200
P	-4.87175900	-0.39423900	-2.79550000
H	-5.12702500	-1.75172500	-2.97083400
H	-5.72226700	-0.10894100	-1.73023900
H	-5.60977500	0.13862700	-3.85173400
I	-2.50223900	-1.46260900	-0.30633100

Model system 4a

MP2 optimization

Ir	-2.57075000	-1.18627400	-1.43792300
Au	-2.58974000	-1.43060100	1.35463800
P	-0.35284300	-0.69920800	-1.52276800
H	0.50952100	-1.58212500	-0.86614000
H	0.18522900	-0.71602000	-2.81370500
H	0.10539500	0.53513900	-1.05528400
C	-3.03222100	0.53016400	-1.14596000
O	-3.33081500	1.63672900	-0.92124600
Cl	-1.98047800	-3.37561800	-1.86586500
P	-4.73194900	-1.85429900	-1.65358000
H	-5.11884700	-2.11508900	-2.97214900
H	-5.08230300	-3.05710000	-1.03318100
H	-5.76425600	-1.01311800	-1.23061900
P	-3.15282900	0.60749500	2.20622200
H	-2.31291000	1.67512600	1.88455100
H	-4.39033800	1.12713900	1.82241000
H	-3.22747200	0.73304100	3.59450400
P	-2.01178400	-3.63272600	1.45371400
H	-2.87481900	-4.52872900	0.82584100
H	-0.78576500	-3.97764500	0.88822200
H	-1.90086500	-4.19737200	2.72736100

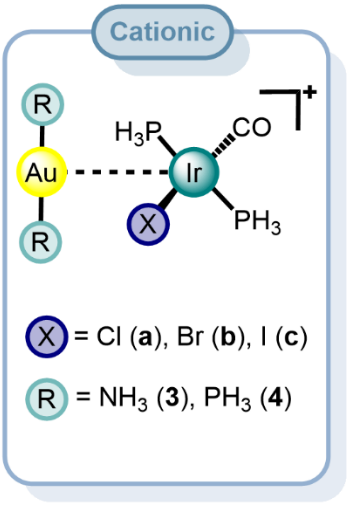

RHF optimization

Ir	-2.86837300	-0.07402500	-2.24276700
Au	-2.21076500	-2.89992400	2.92858200
P	-0.61388000	0.57811600	-2.04543300
H	-0.30025900	1.38717800	-0.95535600
H	0.30730200	-0.45162300	-1.86693000
H	0.00381500	1.29170000	-3.07097900
C	-3.03924800	0.70820200	-3.92755600
O	-3.14141300	1.17575300	-4.93389900
Cl	-2.64271100	-1.10653400	-0.02145900
P	-5.09899000	-0.82988100	-2.24426500
H	-5.27880800	-2.20525300	-2.11457400
H	-5.90471900	-0.37221400	-1.20381000
H	-5.92833300	-0.57055700	-3.33395900
P	-2.93078400	-0.68265800	3.46555000
H	-2.09240900	0.33052600	3.02209100
H	-4.15823400	-0.31806200	2.93029500
H	-3.08975400	-0.36681500	4.81142500
P	-1.49396300	-5.10481100	2.37445800
H	-2.36131800	-5.83798000	1.57250700
H	-0.30147100	-5.19161500	1.66495500
H	-1.26652500	-5.98061200	3.43017000

RI-SCS-MP2-ZORA optimization

Ir	-2.56609672351849	-1.19498439264054	-1.45614356831193
Au	-2.57904553987515	-1.42954366183489	1.37666334172612
P	-0.31571267170066	-0.74972074499653	-1.55318559915420
H	0.54770790749408	-1.64045664112291	-0.89396259796588
H	0.22280763960626	-0.77477492190589	-2.85099532746541
H	0.16627027640488	0.48566793952051	-1.09043810290900
C	-2.98162667485449	0.54209545035066	-1.17101811954682
O	-3.25437364901097	1.65567603076726	-0.95493337163551
Cl	-2.04836115155179	-3.42012422880100	-1.89333749551392
P	-4.76969629370527	-1.80093572566518	-1.65763850020076
H	-5.17532874758081	-2.06817107374255	-2.97620590271275
H	-5.17513125663073	-2.98049403942347	-1.01202640600160
H	-5.77368569969275	-0.90792237699840	-1.24769880736679
P	-3.24406627460936	0.61038116309372	2.23866925445683
H	-2.42045496783507	1.71228190932100	1.96582862335185
H	-4.48600328158382	1.10467544142491	1.81382559364438
H	-3.37740320029447	0.71166102137261	3.63129585045357
P	-1.92547018119374	-3.64322661195610	1.47255624257258
H	-2.79746830890574	-4.56864862595204	0.88770437845413
H	-0.71104995189936	-3.97035693755481	0.85790924485745
H	-1.74665024906249	-4.19416997325634	2.75217426926767

SCS-MP2-ZORA^{NR} optimization

Ir	-2.58374205525049	-1.11472713311085	-1.63598189033256
Au	-2.58980959632745	-1.41657676958115	1.22904573588891
P	-0.27561823321537	-0.64123613356615	-1.67156275933404
H	0.55767559313687	-1.45893782441940	-0.89089817211967
H	0.30688840454192	-0.81822280226528	-2.93798819324401
H	0.22035899089637	0.63035373970647	-1.33665384138482
C	-3.06468166366061	0.67985743424765	-1.38915043679994
O	-3.36086336189036	1.78082690825370	-1.18848798442462
Cl	-1.97569688803716	-3.39768087824995	-1.82565115572717
P	-4.81920220085880	-1.84013307789125	-1.80743547247329
H	-5.16695951658882	-2.26260489980360	-3.10167427612139
H	-5.18320856041087	-2.97376085726170	-1.06255439842293
H	-5.89540602511686	-0.98338947805452	-1.51955570426395
P	-3.19882315982973	0.75165832861106	2.48169811042861
H	-2.38578088963936	1.87754635542616	2.26279338446561
H	-4.44739867079531	1.33395595398742	2.20139525075383
H	-3.24871409925584	0.78223795153721	3.88612675248585
P	-1.95041796282448	-3.88296111506913	1.60139554589418
H	-2.78903423043613	-4.83258928494200	0.99827135318448
H	-0.72047089117159	-4.28697459062016	1.06026136886737
H	-1.83993398326585	-4.44773282693449	2.88564978267957

Model system 4b

MP2 optimization

Ir	-2.44871100	-1.11390800	-1.44036400
Au	-2.71848900	-1.49504200	1.32305400
P	-0.23257500	-0.63477500	-1.29859400
H	0.56668100	-1.52970700	-0.58113300
H	0.42837600	-0.62062400	-2.53095100
H	0.17449200	0.58865600	-0.75906600
C	-2.93844600	0.57902100	-1.05832700
O	-3.25724300	1.66892500	-0.78606500
P	-4.58152500	-1.76196500	-1.87860400
H	-4.84274400	-1.98684200	-3.23394700
H	-5.00441000	-2.97365200	-1.32412600
H	-5.64200600	-0.91889800	-1.53481000
Br	-1.78131300	-3.36897700	-2.06208500
P	-3.35650700	0.48467400	2.25903200
H	-2.49523700	1.56947300	2.08464700
H	-4.55828400	1.03473300	1.80941700
H	-3.54897500	0.51573500	3.64135000
P	-2.15508200	-3.70209400	1.38828200
H	-2.95667800	-4.56995200	0.64852500
H	-0.88251800	-4.03230000	0.92512000
H	-2.16270600	-4.31824400	2.64315000

RHF optimization

Ir	-2.62431700	0.03423600	-2.31178300
Au	-2.54803100	-3.06602800	3.03277300
P	-0.38766900	0.63561200	-1.88034900
H	-0.15938200	1.37721000	-0.72359000
H	0.50521400	-0.41589200	-1.68814100
H	0.31887400	1.39804000	-2.80914900
C	-2.62869900	0.92123700	-3.95330800
O	-2.63142500	1.45109100	-4.93315100
P	-4.85707600	-0.67031800	-2.57406000
H	-5.07367400	-2.04601100	-2.55407400
H	-5.75459000	-0.25768800	-1.59203400
H	-5.56591300	-0.32144600	-3.72254000
Br	-2.61766100	-1.20656100	-0.01886400
P	-3.28652200	-0.88675000	3.68563400
H	-2.40157500	0.14294000	3.39505100
H	-4.45789500	-0.45820800	3.07566200
H	-3.55874100	-0.67138400	5.03292000
P	-1.80902900	-5.22398300	2.33654100
H	-2.61552600	-5.86003000	1.39974800
H	-0.56344800	-5.26074500	1.72030100
H	-1.67681400	-6.20107900	3.31691700

Model system 4c

MP2 optimization

Ir	-2.43979700	-1.15248400	-1.46107000
Au	-2.72263400	-1.45736300	1.30539200
P	-0.22251400	-0.68338100	-1.31481400
H	0.58056900	-1.56982400	-0.59094900
H	0.45142400	-0.65929200	-2.53982700
H	0.17311400	0.54196400	-0.76885100
C	-2.91914000	0.54224500	-1.04952000
O	-3.22905800	1.63418100	-0.77610600
P	-4.58196200	-1.77886500	-1.88126500
H	-4.87247400	-1.99713700	-3.23159500
H	-5.02770900	-2.97912100	-1.31966400
H	-5.62052400	-0.91391400	-1.52166000
I	-1.74065300	-3.52291600	-2.25742500
P	-3.34343300	0.51698800	2.26542700
H	-2.47754000	1.59938400	2.09791800
H	-4.54437300	1.08006200	1.82941800
H	-3.52834300	0.53753700	3.64906700
P	-2.18227900	-3.66875100	1.42293400
H	-2.98924200	-4.54500800	0.69801300
H	-0.91273200	-4.02326400	0.96785700
H	-2.19731600	-4.26358300	2.68874600

RHF optimization

Ir	-2.58262100	-0.01578600	-2.48126000
Au	-2.60117300	-3.02149100	3.28432900
P	-0.34634500	0.60197800	-2.06593500
H	-0.11394600	1.36851300	-0.92685700
H	0.55884000	-0.43694800	-1.86509400
H	0.34251600	1.35316600	-3.01738700
C	-2.57713600	0.84741700	-4.13764200
O	-2.57387500	1.36199900	-5.12501100
P	-4.81911400	-0.70464800	-2.76167600
H	-5.05480000	-2.07686200	-2.73831200
H	-5.72865500	-0.27172200	-1.80022300
H	-5.50075000	-0.35381600	-3.92630700
P	-3.35141500	-0.97641800	4.26518100
H	-2.50077600	0.10765400	4.08605300
H	-4.55484600	-0.49265200	3.76657100
H	-3.57158600	-0.95377300	5.63818700
P	-1.85048100	-5.02634300	2.22502000
H	-2.69194200	-5.51891400	1.23482700
H	-0.63795100	-4.91882900	1.55466600
H	-1.64703400	-6.14757300	3.02217400
I	-2.59080600	-1.31071200	0.00319800

Model system 5a

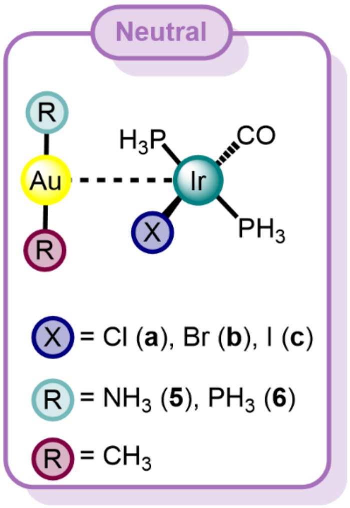

MP2 optimization

Ir	-2.76571800	-1.14273900	-1.30591900
Au	-2.30674600	-1.60297000	1.60267800
P	-0.49823100	-1.10060900	-1.20282200
H	0.11558600	-2.28834100	-0.79915100
H	0.18362900	-0.86118300	-2.40450600
H	0.14231700	-0.16760300	-0.38166000
C	-2.89760500	0.60768000	-0.95736900
O	-2.99821300	1.75272900	-0.70192900
Cl	-2.63579600	-3.38053100	-1.93797600
P	-4.93280200	-1.39116100	-1.80661000
H	-5.19713800	-1.81642000	-3.11204900
H	-5.67511700	-2.34638400	-1.09802000
H	-5.81366400	-0.30432900	-1.72395400
C	-1.15973600	-3.22440600	1.43770700
H	-1.38672200	-3.90488500	2.25922200
H	-1.34818900	-3.73507800	0.49586400
H	-0.10264500	-2.96071600	1.50560700
N	-3.54846300	0.09003800	1.97612000
H	-3.12701900	0.95061900	1.64301200
H	-4.42770000	-0.00869000	1.48217200
H	-3.75802300	0.20391200	2.96110900

RHF optimization

Ir	-3.28250300	-1.40612600	-2.50220400
Au	-1.58344300	-1.14612500	3.08003800
P	-1.34515000	-2.32440900	-1.52428500
H	-1.21157500	-3.70238200	-1.66928500
H	-0.14507100	-1.88797900	-2.07837600
H	-1.07349900	-2.17774200	-0.16558900
C	-3.91755200	-0.67826000	-0.92956200
O	-4.31164900	-0.23152500	0.02437900
P	-5.07623500	-0.64636900	-3.80879900
H	-4.73540300	0.17761000	-4.87811500
H	-5.80077400	-1.63486400	-4.46945700
H	-6.12229000	0.09365800	-3.25370800
C	0.16875800	-2.21835400	2.89246400
H	0.67311500	-2.29898700	3.84958100
H	-0.02083400	-3.22552700	2.53423100
H	0.85292000	-1.73902900	2.19903500
N	-3.46558900	0.00140100	3.26280500
H	-3.29501300	0.92977600	3.59142800
H	-3.91639000	0.06554200	2.37210100
H	-4.09864500	-0.43742300	3.89970500
Cl	-2.46428200	-2.35193800	-4.56395300

RI-SCS-MP2-ZORA optimization

Ir	-2.71132124946000	-1.12616472046699	-1.45001198313940
Au	-2.36153965123857	-1.61287019231628	1.64326852649647
P	-0.43330477041665	-1.05886450514687	-1.22993885660091
H	0.18082702460242	-2.23535101489167	-0.77707132681773
H	0.31178531013653	-0.82318139950173	-2.40256938750796
H	0.15508084439538	-0.10274678627875	-0.38380201692331
C	-2.89234826318313	0.61114161620939	-1.03785617529120
O	-3.02580303415462	1.73983743581140	-0.73215022506432
Cl	-2.49408547806846	-3.36533527087513	-2.09430856756833
P	-4.91594684704988	-1.41914748374198	-1.88460228781286
H	-5.24732480263948	-1.80718901987421	-3.19441992825623
H	-5.59162444652502	-2.42106444794166	-1.16345070761739
H	-5.82395477420611	-0.35584110526534	-1.71619291905606
C	-1.27999320587991	-3.29572637351240	1.48322678424472
H	-1.55169027907688	-3.97673725685987	2.30192402986553
H	-1.47251588565757	-3.79719437421509	0.52802930471403
H	-0.20467056417565	-3.08078955753839	1.57168352804922
N	-3.53941491056173	0.15203246998372	2.05731430617619
H	-3.32947183954840	0.92816592470057	1.43122252617731
H	-4.53347672590120	-0.05030308705884	1.96909270096864
H	-3.37720112139088	0.46626328878022	3.01213973496358

SCS-MP2-ZORA^{NR} optimization

Ir	-2.65130198143618	-1.14744901075851	-1.50730871995759
Au	-2.45016606936149	-1.53737504218626	1.64090273393797
P	-0.30598859746945	-1.08907082523897	-1.30047711156514
H	0.27755988483397	-2.28038583601213	-0.84724647800869
H	0.43743937050892	-0.87793992905598	-2.47843962044799
H	0.32835310795040	-0.15255306809180	-0.46391618022788
C	-2.86761379126827	0.60925507870990	-0.97146931384117
O	-3.02583841755103	1.71201207569796	-0.61694057318286
Cl	-2.39336610861861	-3.36930651364870	-2.33883570448356
P	-4.92372433835978	-1.50000810368905	-1.90533306375801
H	-5.28193710764417	-1.79164972432845	-3.23281771370473
H	-5.50154724747912	-2.60566109974376	-1.25742473227458
H	-5.90484035873028	-0.53004994381721	-1.61682817661287
C	-1.20508196088579	-3.31077153294927	1.41106285690542
H	-1.45510216018111	-4.02634330515289	2.20455735699959
H	-1.39800998828792	-3.77255100225851	0.43666735551790
H	-0.13920553753913	-3.06132794168309	1.50104352236324
N	-3.76422252574918	0.30575504129249	2.24229646525759
H	-3.62320536977832	1.09335957932828	1.61336941775604
H	-4.75696236412659	0.08411083689143	2.23593361418558
H	-3.53323310882677	0.61688440669455	3.18273112514176

Model system 5b

MP2 optimization

Ir	-2.75863900	-1.14151200	-1.29235600
Au	-2.31869900	-1.60965600	1.60167900
P	-0.49052900	-1.11499200	-1.22796300
H	0.13540500	-2.27760400	-0.77282600
H	0.16606000	-0.94078400	-2.45415700
H	0.16630400	-0.13996800	-0.46955400
C	-2.87642700	0.61030600	-0.93581600
O	-2.96808700	1.75568400	-0.68122500
P	-4.93073200	-1.35919100	-1.78122500
H	-5.22037600	-1.72415600	-3.09959300
H	-5.67836800	-2.33248500	-1.10324500
H	-5.79817600	-0.26584100	-1.64305300
C	-1.13679500	-3.20697700	1.45094900
H	-1.36038800	-3.89087900	2.27068200
H	-1.30166900	-3.72409800	0.50845900
H	-0.08694400	-2.91909200	1.53202400
N	-3.59344300	0.06033100	1.97136500
H	-3.16802900	0.93221200	1.67461800
H	-4.45276800	-0.03483100	1.44290500
H	-3.83969500	0.14877800	2.95052700
Br	-2.63072500	-3.48601100	-2.02053400

RHF optimization

Ir	-3.26163700	-1.42469200	-2.53102100
Au	-1.59870100	-1.13560300	3.08818900
P	-1.33430200	-2.32887000	-1.51898800
H	-1.18615800	-3.70822100	-1.62787000
H	-0.11907600	-1.89274900	-2.03806300
H	-1.10966100	-2.15265000	-0.15463100
C	-3.88748800	-0.69953100	-0.94925700
O	-4.27632200	-0.25521000	0.00598900
P	-5.08602300	-0.63366400	-3.77604400
H	-4.78989200	0.21929600	-4.83530300
H	-5.85578200	-1.59426100	-4.42565200
H	-6.09580400	0.10133100	-3.14956400
C	0.15043400	-2.20970400	2.88547000
H	0.66015400	-2.29469800	3.83930900
H	-0.04295100	-3.21515300	2.52455600
H	0.83112500	-1.72859900	2.18992400
N	-3.47848100	0.01460700	3.28776900
H	-3.30418700	0.94122600	3.61945800
H	-3.93465900	0.08347400	2.40028900
H	-4.10824200	-0.42615000	3.92663800
I	-2.34344800	-2.49923100	-4.89876500

Model system 5c

MP2 optimization

Ir	-2.73288500	-1.15491300	-1.26745600
Au	-2.35990400	-1.62135600	1.59803500
P	-0.46606800	-1.15237000	-1.26684900
H	0.18247900	-2.26362700	-0.72192700
H	0.15526900	-1.09278900	-2.52100700
H	0.21221600	-0.11250400	-0.61904600
C	-2.82676500	0.60496900	-0.91906100
O	-2.90401400	1.75385700	-0.67925400
P	-4.91163100	-1.32739500	-1.73823100
H	-5.23976800	-1.62614300	-3.06447700
H	-5.67893600	-2.30568700	-1.08894900
H	-5.75110300	-0.21919700	-1.54093200
C	-1.14238600	-3.19296100	1.47092100
H	-1.38891800	-3.89454700	2.26904900
H	-1.25243300	-3.69995500	0.51555800
H	-0.10457900	-2.88114600	1.60354400
N	-3.66428700	0.03082400	1.95419700
H	-3.23030500	0.90956500	1.69190100
H	-4.50143500	-0.05705200	1.39009100
H	-3.94963500	0.09938700	2.92441400
I	-2.61601200	-3.63601300	-2.12809000

RHF optimization

Ir	-3.26163700	-1.42469200	-2.53102100
Au	-1.59870100	-1.13560300	3.08818900
P	-1.33430200	-2.32887000	-1.51898800
H	-1.18615800	-3.70822100	-1.62787000
H	-0.11907600	-1.89274900	-2.03806300
H	-1.10966100	-2.15265000	-0.15463100
C	-3.88748800	-0.69953100	-0.94925700
O	-4.27632200	-0.25521000	0.00598900
P	-5.08602300	-0.63366400	-3.77604400
H	-4.78989200	0.21929600	-4.83530300
H	-5.85578200	-1.59426100	-4.42565200
H	-6.09580400	0.10133100	-3.14956400
C	0.15043400	-2.20970400	2.88547000
H	0.66015400	-2.29469800	3.83930900
H	-0.04295100	-3.21515300	2.52455600
H	0.83112500	-1.72859900	2.18992400
N	-3.47848100	0.01460700	3.28776900
H	-3.30418700	0.94122600	3.61945800
H	-3.93465900	0.08347400	2.40028900
H	-4.10824200	-0.42615000	3.92663800
I	-2.34344800	-2.49923100	-4.89876500

Model system 6a

MP2 optimization

Ir	-2.74084700	-0.99218400	-1.29977400
Au	-2.40277100	-1.80514800	1.59563800
P	-0.48416100	-1.00810700	-1.06557200
H	0.11985000	-2.25167200	-0.86101900
H	0.26743700	-0.53267100	-2.14957800
H	0.09938200	-0.26155000	-0.03748500
C	-2.79659900	0.79111500	-1.11046700
O	-2.85028500	1.95895400	-1.00766500
P	-4.91296500	-1.17268200	-1.83581000
H	-5.16790300	-1.76068000	-3.07771200
H	-5.72200800	-1.97719200	-1.02163900
H	-5.72190700	-0.03275500	-1.92135100
P	-3.87765500	-0.13906100	1.96407800
H	-5.11018500	-0.16664200	1.29885200
H	-4.35775700	0.08345700	3.26432100
H	-3.50204100	1.17199600	1.65195200
C	-1.07903100	-3.33841900	1.41741800
H	-1.24086600	-3.84555500	0.46648100
H	-0.04618100	-2.98655500	1.47445300
H	-1.23219600	-4.04871200	2.22967200
Cl	-2.73678500	-3.29127900	-1.78750900

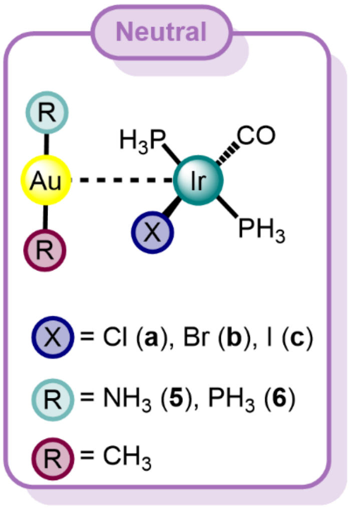

RHF optimization

Ir	-2.97305800	-1.69567200	-2.91265900
P	-0.96017800	-2.74758400	-2.32822900
H	-0.88908600	-4.10995500	-2.60698700
H	0.17372500	-2.29230000	-2.99590800
H	-0.48766400	-2.74269500	-1.01413100
C	-3.34500800	-1.11115900	-1.19732600
O	-3.56415500	-0.75968500	-0.15357500
P	-4.91561500	-0.76262700	-3.86044800
H	-4.69109900	0.14845000	-4.88881200
H	-5.75482800	-1.67096800	-4.49969400
H	-5.83837600	-0.05823500	-3.08947700
Au	-7.15362600	1.35866700	-0.07644000
P	-5.86519200	1.02590500	1.89254600
H	-4.56460000	1.52567400	1.91789400
H	-6.34604700	1.56327300	3.08708700
C	-8.28247200	1.64574900	-1.82238900
H	-8.59160600	0.69890000	-2.25435800
H	-9.17946000	2.21723700	-1.60812300
H	-7.71829000	2.18943500	-2.57391400
H	-5.62365400	-0.28302700	2.30522900
Cl	-2.47746400	-2.46317400	-5.14536800

RI-SCS-MP2-ZORA optimization

Ir	-2.76704987339513	-0.97227995087687	-1.35409419788189
Au	-2.34892427894725	-1.85165202859118	1.63963157062395
P	-0.49403886091364	-1.06232960406896	-1.08906686500637
H	0.05857283830863	-2.33071591617678	-0.86152079843735
H	0.27111879263859	-0.64544902564794	-2.19718789895212
H	0.12765290009231	-0.30303852577666	-0.08258518818897
C	-2.79109568355333	0.82052960988072	-1.15526670135493
O	-2.82097289706515	1.98627417589614	-1.03889527247249
Cl	-2.75414688034737	-3.26648236570243	-1.79081800490110
P	-4.96205599413781	-1.08775936564704	-1.91147770487644
H	-5.23508891977600	-1.52225051212074	-3.21985623336472
H	-5.78283728528846	-1.97662462232384	-1.19121765208689
H	-5.76926392439125	0.06555934279933	-1.86876187020910
P	-3.90306434018979	-0.17940904429067	1.91652909234807
H	-5.14209858706498	-0.26979685354458	1.25461773397156
H	-4.39387675155410	0.06827883031669	3.21615176764295
H	-3.56723716850427	1.13945708172569	1.56461949751320
C	-0.99026632593285	-3.37526191735577	1.59860551569246
H	-1.13419528934400	-3.98310517087832	0.69681913694349
H	0.04365696377987	-3.00212003693702	1.62249039914085
H	-1.13323509001520	-4.01397095079790	2.48061159088983

SCS-MP2-ZORA^{NR} optimization

Ir	-2.77438252495175	-0.87424791164453	-1.37141654756685
Au	-2.34343492724453	-1.99102067448091	1.58467808365729
P	-0.45482590432714	-1.22859631512457	-1.13787282975533
H	-0.04206392605160	-2.55618435725069	-0.96709918169923
H	0.30563986310172	-0.86518880425803	-2.26892798667256
H	0.29407900009394	-0.57421053568945	-0.14549582741295
C	-2.61106647826000	0.95379055525589	-1.05995788516544
O	-2.53083587421450	2.09571303000983	-0.85287640523215
Cl	-3.02344263761135	-3.20195671587364	-1.80689677782646
P	-5.02605859714559	-0.75343448411446	-1.98851432796126
H	-5.27202622527897	-1.03067833061183	-3.34431390796579
H	-5.91724718329807	-1.66856039876517	-1.39967890025602
H	-5.78961944394212	0.42458187626658	-1.86044128663603
P	-4.29172305609134	-0.43434810086916	1.92083946345873
H	-5.48223256131547	-0.60542501886383	1.18658266041239
H	-4.89274901501334	-0.27510531657214	3.18864096410164
H	-4.09187238120015	0.93079419222480	1.63949475459358
C	-0.60637153150618	-3.33832646409202	1.69742590950022
H	-0.66645639971229	-4.06914748973877	0.88022862990910
H	0.35339262771125	-2.80825330039354	1.63126358607163
H	-0.62039514374231	-3.88391137541427	2.64933043244549

Model system 6b

MP2 optimization

Ir	-2.80380100	-0.98772000	-1.26139200
Au	-2.32813200	-1.84836200	1.56979600
P	-0.54819400	-1.18073000	-1.12039300
H	-0.00612800	-2.43359200	-0.83031800
H	0.14026000	-0.87267500	-2.30289100
H	0.15342800	-0.36485000	-0.22604100
C	-2.72024300	0.80255700	-1.05231500
O	-2.68451900	1.96739400	-0.93244700
P	-4.96639000	-0.93615500	-1.84192900
H	-5.25710400	-1.31226000	-3.15694300
H	-5.88096700	-1.75358200	-1.16130400
H	-5.66134700	0.28109400	-1.78305200
P	-4.00515300	-0.36355000	1.82188700
H	-5.22123400	-0.54830300	1.15291500
H	-4.52684700	-0.19253200	3.11584700
H	-3.78183100	0.98186400	1.51101100
C	-0.82256500	-3.21964800	1.59826500
H	-0.91312600	-3.90320300	0.75532800
H	0.15965800	-2.74640500	1.57218400
H	-0.88638900	-3.79716600	2.52059500
I	-2.95617500	-3.53387900	-1.84290700

RHF optimization

Ir	-2.97172400	-1.70006200	-2.92833800
P	-0.96201500	-2.74345300	-2.31672500
H	-0.88032900	-4.10844700	-2.57785700
H	0.18233600	-2.28957200	-2.96674400
H	-0.51461500	-2.72196400	-0.99366100
C	-3.34768600	-1.11377400	-1.21357900
O	-3.56850000	-0.76186600	-0.17111100
P	-4.91924700	-0.76178100	-3.86163600
H	-4.70667600	0.15805500	-4.88438300
H	-5.77020400	-1.66261000	-4.49541500
H	-5.82930900	-0.06139600	-3.07169400
Au	-7.15303700	1.36069100	-0.06720200
P	-5.86984300	1.03129500	1.90568800
H	-4.56921800	1.53113900	1.93323400
H	-6.35294800	1.56979100	3.09876100
C	-8.27688400	1.64454400	-1.81678800
H	-8.58483500	0.69697000	-2.24793900
H	-9.17443200	2.21634900	-1.60581800
H	-7.71072600	2.18700000	-2.56767300
H	-5.62868300	-0.27716700	2.32041000
Br	-2.43917900	-2.51753500	-5.29660700

Model system 6c

MP2 optimization

Ir	-2.80380100	-0.98772000	-1.26139200
Au	-2.32813200	-1.84836200	1.56979600
P	-0.54819400	-1.18073000	-1.12039300
H	-0.00612800	-2.43359200	-0.83031800
H	0.14026000	-0.87267500	-2.30289100
H	0.15342800	-0.36485000	-0.22604100
C	-2.72024300	0.80255700	-1.05231500
O	-2.68451900	1.96739400	-0.93244700
P	-4.96639000	-0.93615500	-1.84192900
H	-5.25710400	-1.31226000	-3.15694300
H	-5.88096700	-1.75358200	-1.16130400
H	-5.66134700	0.28109400	-1.78305200
P	-4.00515300	-0.36355000	1.82188700
H	-5.22123400	-0.54830300	1.15291500
H	-4.52684700	-0.19253200	3.11584700
H	-3.78183100	0.98186400	1.51101100
C	-0.82256500	-3.21964800	1.59826500
H	-0.91312600	-3.90320300	0.75532800
H	0.15965800	-2.74640500	1.57218400
H	-0.88638900	-3.79716600	2.52059500
I	-2.95617500	-3.53387900	-1.84290700

RHF optimization

Ir	-2.96962800	-1.70694200	-2.95274400
P	-0.96543900	-2.73663800	-2.30028900
H	-0.86692600	-4.10528000	-2.53401700
H	0.19476100	-2.28531300	-2.92298900
H	-0.55570300	-2.69032700	-0.96523100
C	-3.35138600	-1.11756700	-1.23756600
O	-3.57448200	-0.76482000	-0.19678200
P	-4.92483900	-0.76079900	-3.86271700
H	-4.73170800	0.17264000	-4.87667400
H	-5.79438300	-1.64933000	-4.48811200
H	-5.81524100	-0.06692300	-3.04450700
I	-2.38801800	-2.59049300	-5.49995600
Au	-7.15119700	1.36276200	-0.05588400
P	-5.87452100	1.03680500	1.92171300
H	-4.57394100	1.53688100	1.95228500
H	-6.36046800	1.57607200	3.11323100
C	-8.26874500	1.64299900	-1.80995600
H	-8.57732200	0.69458900	-2.23877200
H	-9.16570600	2.21734300	-1.60355900
H	-7.69918800	2.18179100	-2.56088400
H	-5.63367400	-0.27124200	2.33832900
